# ﻿The *Vitextrifolia* complex (Lamiaceae) in the Philippines

**DOI:** 10.3897/phytokeys.248.120387

**Published:** 2024-10-22

**Authors:** Renerio P. Gentallan Jr., Seda Sengun, Michael Cedric B. Bartolome, Kristine Joyce O. Quiñones, Nadine B. Coronado, Teresita H. Borromeo, Emmanuel Bonifacio S. Timog

**Affiliations:** 1 Institute of Crop Science, College of Agriculture and Food Science, University of the Philippines Los Baños, Los Baños, Laguna, Philippines University of the Philippines Los Baños Los Baños Philippines; 2 Institute of Biological Sciences, College of Arts and Sciences, University of the Philippines Los Baños, Los Baños, Laguna, Philippines University of the Philippines Los Baños Los Baños Philippines; 3 Department of Forest Biological Sciences, College of Forestry and Natural Resources, University of the Philippines Los Baños, Los Baños, Laguna, Philippines University of the Philippines Los Baños Los Baños Philippines

**Keywords:** Labiatae, lagundi, Malesia, Subfamily Viticoideae, taxonomy

## Abstract

The *Vitextrifolia* complex in the Philippines comprises economically important medicinal species, but its taxonomic status has become ambiguous due to numerous historical nomenclatural revisions. We assembled the complete chloroplast genomes of five species belonging to this species complex to provide additional evidence for their species delimitation. Based on a detailed analysis of specimens which combined molecular and morphological data, we propose reinstating *V.elmeri* Moldenke and delineating *V.arvensis* Gentallan, Sengun & M.B.Bartolome as a new endemic species belonging to this complex. The new species is a putative hybrid between *V.bicolor* Willd. and *V.elmeri* Moldenke. The specific epithet *arvensis* reflects its predominantly cultivated nature, both on a commercial scale and in home gardens, as a valued medicinal plant. We also provided a key to identify the five species belonging to the *V.trifolia* complex in the Philippines.

## ﻿Introduction

The genus *Vitex* was identified to be the most problematic group among the ‘troublesome’ mints (Bramley et al. 2009). Within this group, the *V.trifolia* complex was first proposed by R.P.J de Kok in 2004 at the Flora Malesiana Symposium (Sengun 2017) which consisted of six taxa, namely *V.agnus-castus* L., *V.benthamiana* Domin, *V.negundo* L., *V.pseudonegundo* (Hausskn. ex Bornm.) Hand.-Mazz., V.trifoliaL.subsp.trifolia and V.trifoliasubsp.littoralis Steenis. It was defined by de Kok (2007) as a group with white waxy undersurface of the leaves, attributed to their synapomorphic character of possessing conical apical cells of the hairs covered with wax plates. Its species are distributed from Greece to China and Japan, through Malesiana to Australia and the Pacific. The species complex was studied by Sengun (2017) for her doctorate, and the resulting treatment reinstated *V.bicolor* Willd., elevated V.trifoliasubsp.trifolia and *littoralis* to species level as *V.trifolia* and *V.rotundifolia*, respectively, and split *V.negundo* s.l. into *V.negundo* s.s., *V.hybrida* Moldenke, and *V.collium* Sengun (Sengun et al. 2024).

Historically, taxa within the *Vitextrifolia* complex have often been confused with each other, resulting in numerous taxonomic treatments and nomenclatural revisions (Lamarck 1786; Lam 1919; Moldenke 1957; Munir 1987; de Kok 2007, 2008; de Kok and Sengun 2020; Sengun et al. 2024). For example, Lam (1919) placed *V.bicolor* as a variety under *V.negundo* and placed *V.rotundifolia* under the synonymy of *V.trifolia*. Moldenke (1957) later revised this and accepted *V.bicolor* and *V.rotundifolia* as varieties of *V.trifolia*. However, van Steenis (1957) elevated *V.rotundifolia* to subspecies level under the name V.trifoliasubsp.littoralis. De Kok (2008) placed *V.bicolor* under V.trifoliasubsp.trifolia and recognized V.trifoliasubsp.littoralis due to differences in growth habit and distribution. Also, adding to the confusion, some of the taxa have been used as medicinal plants since ancient times and have been carried around and cultivated in several parts of the world, particularly in Asia (Sengun 2017).

In the Philippines, there are nine recorded *Vitex* species. Out of which, four species that belong to the *V.trifolia* complex have been documented: *V.bicolor*, *V.negundo*, *V.rotundifolia*, and *V.trifolia* (CDFP 2023), with only *V.negundo* recognized as introduced (POWO 2023). Throughout the taxonomic revisions of this species complex, several taxa originating from the Philippines, such as *V.elmeri* Moldenke, *V.leucoxylon* Blanco, and V.negundovar.philippinensis Moldenke, have been considered heterotypic synonyms (de Kok 2007, 2008; Sengun 2017; Sengun et al. 2024). The *Vitextrifolia* complex holds economic significance in the Philippines. Three of the four species, which occur in the country, are collectively referred to as “lagundi” (Madulid 2001). These are cultivated not only by people but also by the commercial pharmaceutical companies. The production of lagundi-based products in the Philippines was estimated to reach ₱430 million in gross sales and revenues (PCHRD 2010, as cited in Olivar et al. 2016).

Due to numerous taxonomic revisions within the complex and the same common name being applied to the different taxa, some published research data on “lagundi” in the Philippines has become ambiguous, particularly those lacking an associated type specimen and/or description (Manalo 1982; Dayrit et al. 1987; Cantoria 1989; Tadeo 2011). Moreover, upon revisiting the types of *V.negundo* in the Philippine Pharmacopeia 1 (PP1 2004), we observed that the herbarium specimens considered as types can be attributed to two species in the most recent taxonomic treatment. Upon further examination of all specimens within the complex, certain morphological characteristics of the revisited specimens exhibited notable and significant differences from the descriptions of the currently accepted taxa. Specifically, the leaf undersurface of some *V.bicolor* morphotypes from the Philippines do not dry white, resulting in the absence of a distinct color disparity (“bicolor” effect) between the abaxial and adaxial leaf surfaces in herbarium specimens. Additionally, *V.negundo* specimens from the Philippines have entire leaflets at maturity, contrary to the amended description of this species, which have some or all dentate leaflets throughout its life cycle (Sengun et al. 2024).

Given these observations, we aimed to elucidate the phylogenetic position and species delimitation of the taxa within the *Vitextrifolia* complex in the Philippines.

## ﻿Methods

### ﻿Operational Taxonomic Units (OTUs)

For the analysis of variance and molecular phylogeny, all specimens were assigned to eight a priori groups according to the overall pattern of morphology based on the initial observed differences. These Operational Taxonomic Units (OTUs) include:

1. *V.agnus-castus*

2. *V.bicolor* A: *V.bicolor* morphotype A; *V.bicolor* sensu Sengun et al. (2024); entire leaflets with densely pubescent undersurface drying white

3. *V.bicolor* B: *V.bicolor* morphotype B; leaflets mostly dentate when young, occasionally entire, undersurface sparsely pubescent not drying white; inflorescence not regularly dichasially branching (compared to *V.bicolor* A)

4. *V.bicolor* C: *V.bicolor* morphotype C; same with *V.bicolor* B but with petiolulate terminal and lateral leaflets

5. *V.negundo* A: *V.negundo* morphotype A; *sensu*Sengun et al. (2024); always with some dentate leaflets at maturity; Chinese taxon

6. *V.negundo* B: *V.negundo* morphotype B; leaflet margins entire at maturity; morphotypes from the Philippines

7. *V.trifolia*

8. *V.rotundifolia*

These OTUs were selected to represent all the clades of the currently accepted species under the *Vitextrifolia* complex (POWO 2023; WFO 2023).

### ﻿Chloroplast genome and nrDNA assembly

The DNA samples were extracted using a slightly modified CTAB protocol of Doyle and Doyle (1987), and were sent to NovogeneAIT Genomics Singapore PTE LTD, Singapore, for sequencing using the HiSeq-PE150 platform (Illumina Inc., San Diego, CA, USA). NEBNext® Ultra™ II DNA Library Prep Kit (Cat No. E7645) was used for library preparation. Fastp version 0.20.0 (Chen et al. 2018) was used for read-quality control. This generated cleaned reads of approximately 2 Gbp. We sequenced and assembled 16 accessions comprising of 9 operational taxonomic units of *Vitex* (Table 1). The chloroplast genomes and embryophyta plant nuclear ribosomal DNA (18S-ITS1-5.8S-ITS2-26S) sequences were assembled using GetOrganelle v1.7.5+ (Jin et al. 2020). The circularized genomes were then annotated and mapped using GeSeq (Tillich et al. 2017) and CPGAVAS2 (Shi et al. 2019). The annotated plastomes were visualized using OGDRAW (Greiner et al. 2019). The assembled chloroplast genomes and the ITS sequences were submitted to GenBank while other data sources were uploaded at Dryad (https://doi.org/10.5061/dryad.jq2bvq8h1). IRScope (Amiryousefi et al. 2018) was used to visualize the differences in the plastomes, particularly the expansion of inverted repeat (IR) regions at the junction sites among the assembled *Vitex* chloroplast genomes.

**Table 1.** Accessions sequenced for chloroplast genome assembly.

OTU	Plastome GenBank Accession No.*	ITS GenBank Accession No.	Voucher Information	Accession Number	Country
*V.agnus-castus*	PP584505	PP583618	Chase 22221	Kew DNA I.D. 44903	Unknown
*V.bicolor* A	NC_65871.1	PP583617	Gentallan & Bartolome 674	PBN 2018-674	Philippines
*V.bicolor* B	-	PP583616	Gentallan & Bartolome 879	PBN 2019-138	Philippines
		PP583605	Gentallan & Bartolome 1360	PBN 2019-619	Philippines
*V.bicolor* C	-	PP583612	Gentallan & Bartolome 874	PBN 2019-133	Philippines
		PP583607	Gentallan & Bartolome 1545	PBN 2023-007	Philippines
*V.negundo* A	PP584504	PP577658	Sengun 31	ICROPS 1443	China
*V.negundo* B	-	PP583606	Gentallan & Bartolome 918	PBN 2019-177	Philippines
		PP583609	Gentallan & Bartolome 132	PBN 2018-132	Philippines
		PP583610	Gentallan & Bartolome 146	PBN 2018-146	Philippines
		PP583608	Gentallan & Bartolome 116	PBN 2018-116	Philippines
		PP583613	Gentallan & Bartolome 1560	PBN 2023-022	Philippines
		PP583611	Gentallan & Bartolome 1036	PBN 2019-295	Philippines
*V.rotundifolia*	OQ942922.1	PP583604	Gentallan & Bartolome 1537	PBN 2021-033	Philippines
*V.trifolia*	NC_65871.1	PP583615	Gentallan & Bartolome 1073	PBN 2019-332	Philippines
*V.parviflora* (sister group)	ON597620.1	PP583619	Gentallan & Bartolome 1538	ICROPS 1373	Philippines

* other source data not uploaded in GenBank are provided at Dryad.

### ﻿Phylogenetic analysis

The ingroup, sister group, and outgroup were pre-identified using the updated tribal classification of Lamiaceae based on plastome phylogenomics (Zhao et al. 2021). Based on sequence availability, chloroplast genome sequences of five other *Vitex* species (assembled and utilized by Zhao et al. 2021) as part of the ingroup and sister group, one *Congea* species and one *Sphenodesme* species of the closely related subfamily Symphorematoideae Briq. (assembled and utilized by Zhao et al. 2021), and three *Salvia* species of the subfamily Nepetoideae as part of the outgroup were downloaded from the NCBI database. The whole chloroplast genome of downloaded sequences, together with the assembled chloroplast genome sequences of the nine *Vitex* species, were aligned using MAFFT (Katoh and Standley 2013). Using MEGA-X software (Kumar et al. 2018), the best model for phylogenetic analysis using Bayesian information criterion (BIC) was identified, and subsequently a Maximum Likelihood (ML) tree was generated (Nei and Kumar 2000) with 1,000 bootstraps. BI analyses using Markov chain Monte Carlo (MCMC) methods (Yang and Rannala 1997) were performed with MrBayes v3.2.6 (Huelsenbeck and Ronquist 2001) and implemented using Geneious Prime 2022.2.2. For each Bayesian analysis, four MCMC chains were run simultaneously for 2 million generations. Each run began with one random tree and sampled one tree every 1,000 generations. At the end of the run, chain convergence and estimated sample size (ESS) parameters were assessed. In the resulting summary tree, posterior probability values (PP) ≥ 0.95 were considered to be strongly supported (Suzuki et al. 2002).

To achieve the same polarity and congruence with the plastome phylogenetic analysis, the 16 assembled nuclear DNA (nrDNA) sequences were aligned to the ITS sequences available at NCBI representing the phylogenetic groupings. After alignment trimming, the same approach to phylogenetic analysis was performed using MEGA-X and Geneious Prime 2022.2.2.

### ﻿Morphological characterization and examination of herbarium specimens

Extensive fieldwork was conducted to examine living materials across the Philippines. Over 2,000 specimens of the *Vitextrifolia* complex, including types, were examined from various herbaria, namely B, BKF, BM, BO, BR, CAHUP, F, FOF, ICROPS, K, L, LINN, MO, NY, P, PNH, PUH, TAI, TFRI, S, SING, U, UC, and US. Following the recent taxonomic revision of the *Vitextrifolia* complex by Sengun et al. (2024), morphological observations and measurements were collected. Selected portions of the accessions were photographed using the Nikon COOLPIX S9500 Digital Camera and Olympus SZX7 stereomicroscope. Morphological analyses were then conducted using herbarium specimen measurements, following the methodology employed by Sengun et al. (2024) for species delineation within the *V.trifolia* complex. For the morphometric analysis of quantitative data measured from the specimens, our herbarium measurements were augmented with those obtained by Sengun et al. (2024) to provide a more comprehensive representation for the Operational Taxonomic Units (OTUs) involved. We examined 42 quantitative traits (Table 2). The sixth edition of the Royal Horticultural Society color chart was used to characterize color (RHS Media 2015).

**Table 2.** Quantitative characters examined.

TRAIT ACRONYM	TRAIT DESCRIPTION	UNIT
**txl**	longest fully unfolded terminal leaflet length	cm
**txw**	widest fully unfolded terminal leaflet width	cm
**txlwr**	ratio of txl to txw	–
**tml**	shortest fully unfolded terminal leaflet length	cm
**tmw**	narrowest fully unfolded terminal leaflet width	cm
**tmlwr**	ratio of tml to tmw	–
**tpx**	terminal petiolule maximum length	cm
**tpm**	terminal petiolule minimum length	cm
**sxl**	longest fully unfolded lateral leaflet length	cm
**sxw**	widest fully unfolded lateral leaflet width	cm
**sxlwr**	ratio of sxl to sxw	–
**sml**	shortest fully unfolded lateral leaflet length	cm
**smw**	narrowest fully unfolded lateral leaflet width	cm
**smlwr**	ratio of sml to smw	–
**spx**	lateral petiolule maximum length	cm
**spm**	lateral petiolule minimum length	cm
**bxl**	longest fully unfolded basal leaflet length	cm
**bxw**	widest fully unfolded basal leaflet width	cm
**bxlwr**	ratio of bxl to bxw	–
**bml**	shortest fully unfolded basal leaflet length	cm
**bmw**	narrowest fully unfolded basal leaflet width	cm
**bmlwr**	ratio of bml to bmw	–
**bpx**	basal petiolule maximum length	cm
**bpm**	basal petiolule minimum length	cm
**px**	petiole length (maximum)	cm
**pm**	petiole length (minimum)	cm
**cal**	calyx length	mm
**tl**	calyx tooth length	mm
**tw**	calyx tooth width	mm
**tlwr**	calyx tooth length-to-width ratio	–
**cor**	corolla length	mm
**lipl**	lower lip length	mm
**lipw**	lower lip width	mm
**llwr**	lower lip length-to-width ratio	–
**sty**	style length	mm
**stig**	stigma length	mm
**fil**	filament length	mm
**ax**	inflorescence axis length	cm
**frl**	fruit length	mm
**frw**	fruit width	mm
**flwr**	fruit length-to-width ratio	–
**calcov**	percentage calyx coverage	–

### ﻿Principal component analysis (PCA), cluster analysis and visualization

From the 42 quantitative traits examined, principal component analysis was performed using a correlation matrix to effectively summarize the variations observed in the morphological characters using the built-in R function princomp(), and the packages “FactoMineR” (Le et al. 2008) and “factoextra” (Kassambara and Mundt 2017) in R version 4.3.1. All missing data for the principal component and cluster analyses were estimated using the nearest-neighbor approach. A biplot was constructed to visually represent the contributions of original morphological variables to the principal components and the scores of individual specimens on these components to visualize distributions and identify potential patterns or clusters. Additionally, a mean point and confidence ellipse were elucidated per OTU in the biplot. For cluster analysis, the dataset underwent standardization to ensure equal weighting of variables and mitigate measurement error. A dendrogram was created using Ward’s method for cluster analysis using Euclidean distance, and this was cut into clusters based on the number of clusters that minimizes entropy while maintaining meaningful separation between clusters through XLSTAT 2016.

### ﻿Linear discriminant analysis (LDA)

To validate further the clustering of the accessions, the untransformed data of the resultant clusters were evaluated using LDA. All observations with missing data were removed. Percent correct classification rates between clusters were used to gauge the accuracy of discriminating morphology based on the clusters. Pairwise comparisons using Fisher distance and P-values were calculated at 5% level of significance. LDA was implemented using XLSTAT 2016 (Addinsoft, Inc.).

### ﻿Univariate parametric and non-parametric statistical analyses

To reveal significant differences between the means of distinct morpho-clusters across all examined taxa, univariate statistical analyses were conducted. All records with missing data were omitted from the analysis. Prior to parametric statistical testing using analysis of variance (ANOVA), the Shapiro-Wilk test for normality and Levene’s test for homogeneity of variance were applied. Once the assumptions were satisfied, ANOVA was performed, followed by a pairwise mean comparison test using the Honest Significant Difference (HSD) method at a 5% level of significance. For variables that did not meet the assumptions of ANOVA, the non-parametric Kruskal-Wallis test was employed, followed by a multiple pairwise comparison test using Dunn’s test with Bonferroni correction at the same level of significance. Calculations were carried out using XLSTAT 2016 (Addinsoft, Inc.). Quantitative measurements were represented as the mean ± standard error of the mean. Violin graphs were presented as visual support for the identified traits that exhibited significant differences among the morpho-clusters tested.

## ﻿Results and discussion

### ﻿Chloroplast genome assembly and comparison

We had successfully assembled plastomes of the 16 accessions of our eight OTUs and one taxon from the sister group, *Vitexparviflora* (Table 3). These are the first de novo assembled and taxonomically verified chloroplast genomes for *V.agnus-castus*, *V.bicolor*, *V.negundo*, *V.rotundifolia*, and *V.trifolia*. The elucidated chloroplast genomes served as taxonomic references to clarify the molecular phylogenetic position of the *V.trifolia* complex accessions from the Philippines.

**Table 3.** Structural differences in chloroplast genome of the OTUs sequenced.

OTU	IDENTIFICATION NO.	PLASTOME		LSC		SSC		IR		Genes	mRNA	rRNA	tRNA
		bp	GC%	bp	GC%	bp	GC%	bp	GC%				
*Vitexbicolor* A	PBN 2018-674	154460	38.3	85158	36.4	17928	32.7	25687	43.3	135	91	8	36
*Vitexbicolor* B (1)	PBN 2019-138	154460	38.3	85158	36.4	17928	32.7	25687	43.3	135	91	8	36
*Vitexbicolor* B (2)	PBN 2019-619	154460	38.3	85158	36.4	17928	32.7	25687	43.3	135	91	8	36
*Vitexbicolor* C (1)	PBN 2023-007	154460	38.3	85158	36.4	17928	32.7	25687	43.3	135	91	8	36
*Vitexbicolor* C (2)	PBN 2019-133	154460	38.3	85158	36.4	17928	32.7	25687	43.3	135	91	8	36
*Vitextrifolia*	PBN 2019-332	154444	38.3	85148	36.4	17922	32.7	25687	43.3	135	91	8	36
*Vitexrotundifolia*	PBN 2021-033	154446	38.3	85134	36.4	17938	32.7	25687	43.3	135	91	8	36
*Vitexnegundo* A	ICROPS 1443	154496	38.3	85196	36.4	17920	32.8	25690	43.3	135	91	8	36
*Vitexnegundo* B (1)	PBN 2018-132	154491	38.2	85176	36.4	17941	32.7	25687	43.3	135	91	8	36
*Vitexnegundo* B (2)	PBN 2018-116	154486	38.2	85170	36.4	17942	32.7	25687	43.3	135	91	8	36
*Vitexnegundo* B (3)	PBN 2018-146	154492	38.2	85177	36.4	17941	32.7	25687	43.3	135	91	8	36
*Vitexnegundo* B (4)	PBN 2018-295	154490	38.2	85176	36.4	17940	32.7	25687	43.3	135	91	8	36
*Vitexnegundo* B (5)	PBN 2023-022	154479	38.2	85165	36.4	17940	32.7	25687	43.3	135	91	8	36
*Vitexnegundo* B (6)	PBN 2019-177	154475	38.3	85161	36.4	17940	32.7	25687	43.3	135	91	8	36
*Vitexagnus-castus*	Kew DNA ID 44903	154495	38.3	85200	36.4	17915	32.7	25690	43.3	135	91	8	36
*Vitexparviflora*	ICROPS 1373	154024	38.3	84848	36.5	17852	32.9	25662	43.3	137	91	10	36

**Figure 1.**
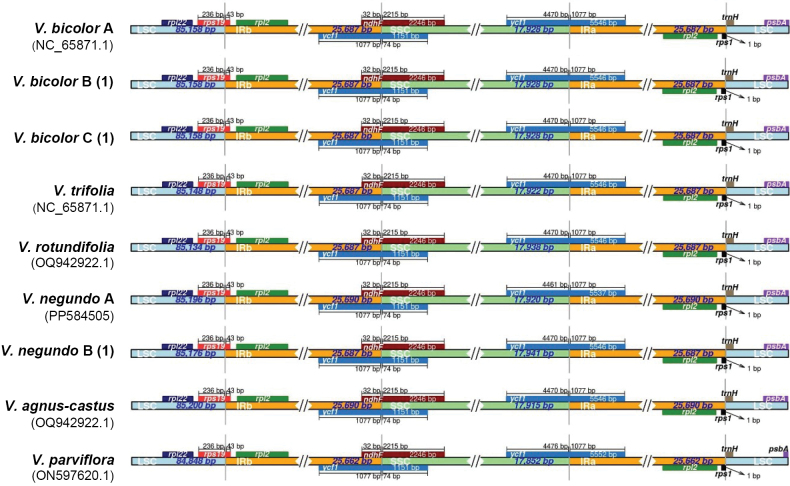
Chloroplast genome comparative analysis of assembled *Vitex* genomes using IRscope. JLB/JLA means the junction between LSC and IRb/IRa, and JSB/JSA means the junction between SSC and IRb/IRa.

The lengths of the plastomes ranged from 154,024 bp (*V.parviflora*) to 154,496 bp (*V.negundo* A). The organization of the four distinct junction sites, including the number of genes and the GC% of the IR and LSC regions, of the chloroplast genome was conserved across the plastomes assembled (Fig. 1). However, we observed significant differences in the length of the IR region between *V.negundo* B and *V.negundo* A. The IR regions of *V.negundo* B, *V.rotundifolia*, *V.trifolia*, and *V.bicolor* were all of equal length, while *V.negundo* A exhibited the same length in this region as *V.agnus-castus* (Table 3; Fig. 1). Although the difference is merely three base pairs, this finding indicated evolutionary divergence of *V.negundo* B and *V.negundo* A, as the region displayed a more conserved nature among the regions of the plastomes examined. A similar pattern of differences persisted in the LSC and SSC regions, although intra-OTU differences existed. On the other hand, unlike *V.negundo* B which showed plastomic differences, *V.bicolor* A, B and C were observed to have the same plastome structure despite being derived from different type localities (Table 3; Fig. 1).

### ﻿Chloroplast genome phylogenetic analysis

In the plastome-based cladogram, two major clades, the *Vitextrifolia* complex and other *Vitex* species, were elucidated with strong maximum likelihood bootstrap support (MLBS) of 100% and Bayesian information posterior probability (BIPP) of 1.00, further validating the monophyletic nature of the *V.trifolia* complex. Within the OTUs examined, *V.agnus-castus* was the earliest diverging lineage, followed by *V.negundo* A. Most of the OTUs, except for *V.bicolor* B and C, received maximal support values (MLBS > 94% and BIPP = 1.00), indicating significant differentiation among the six a priori groups.

**Figure 2.**
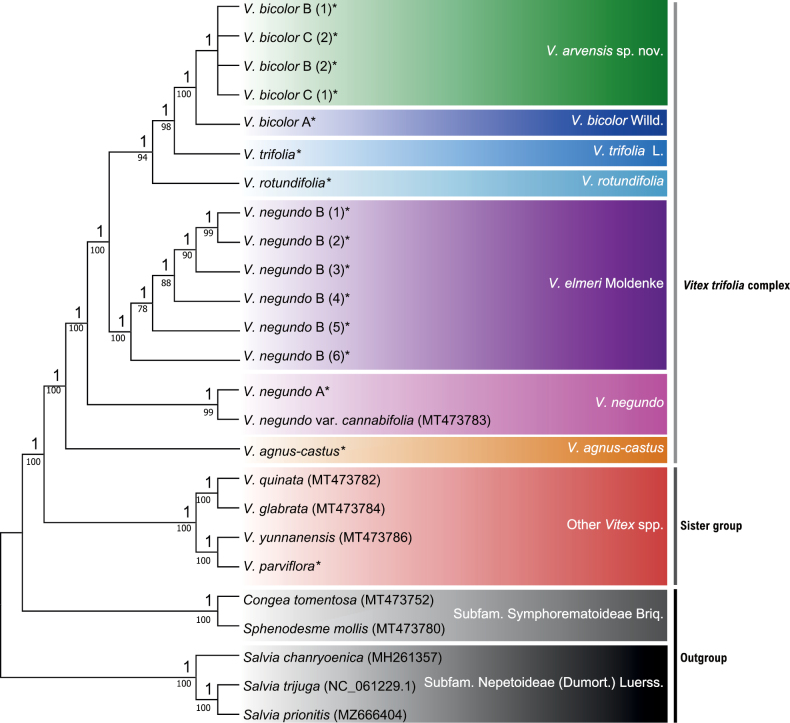
Phylogenetic consensus tree (GTR+G+I model) from the Bayesian inference on the whole chloroplast genome dataset. Numbers above branches indicate posterior probability from the BI analysis, below by bootstrap values for the ML analysis; accessions assembled by authors in asterisk (*).

With 100% bootstrap support, the *V.negundo* B accessions from the Philippines exhibited a closer phylogenetic relationship to *V.rotundifolia*, *V.trifolia*, and *V.bicolor*. The phylogenetic tree suggested that the collective *V.negundo* B specimens examined were evolutionarily divergent from the *V.negundo* A (Fig. 2), supporting the initial structural differences observed during plastome comparison (Fig. 1). This provided compelling evidence that the *V.negundo* B from the Philippines belongs to a different taxonomic group. At the very least, they have maternal progenitors that are evolutionarily divergent from the *V.negundo* A type from China. Our de novo assembled *V.negundo* A (*Sengun* 31) showed a close relationship to a GenBank accession of V.negundovar.cannabifolia (MT473783.1), now a heterotypic synonym of *V.negundo*, which was utilized in updating the tribal classification of Lamiaceae based on plastome phylogenomics (Zhao et al. 2021). Hence, the plastome phylogenetic analysis, along with the plastome structural variations, supported a separate species delineation for the Philippine *V.negundo* B populations from *V.negundo* A.

On the other hand, the representative morphotypes of *V.bicolor* (A, B and C) formed a single clade, indicating that each of the *V.bicolor* accessions was closely related. However, we observed that *V.bicolor* A showed complete divergence from *V.bicolor* B and *V.bicolor* C with 1.00 BIPP and 100% MLBS (Fig. 2). Within the clades of *V.bicolor* B and *V.bicolor* C types, there was low bootstrap support (<64%), suggesting that these two types may belong to a single taxonomic unit. However, based on the plastome phylogenetic analysis, we cannot rule out the possibility that *V.bicolor* B and *V.bicolor* C are simply infraspecies genotypic variants of *V.bicolor*; thus, nrDNA-based phylogenetic analysis was done.

### ﻿nrDNA phylogenetic analysis

**Figure 3.**
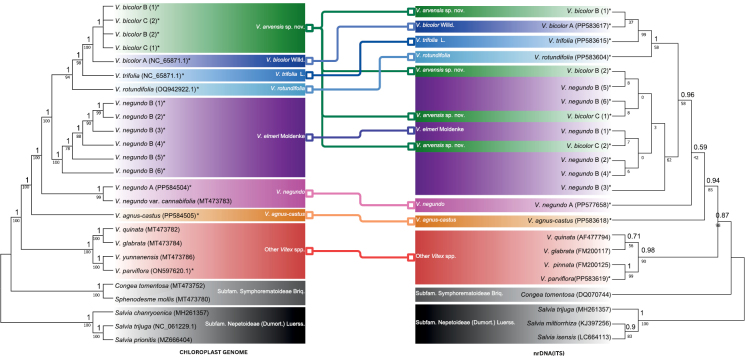
Tanglegram showing discordance between plastome and nrDNA cladograms. Numbers above branches indicate posterior probability from the BI analysis, below by bootstrap values for the ML analysis; accessions assembled by authors in asterisk (*).

The total length of the ITS alignments was 604 bp, comprising 205 variable sites (33.94%) and 120 parsimony-informative characters (PICs; 19.87%). Remarkably, a similar topology was elucidated when compared to the plastome-based phylogram, albeit with lower support values (Fig. 3). Consistent topology and comparably low support were observed with earlier phylogenies within this complex using the same marker (Bramley et al. 2009; Sengun 2017). A notable difference was the polyphyletic nature of *V.bicolor* B and C, resulting in a non-congruent topology in the nrDNA-based cladogram. We suspect this incongruence to be a consequence of interspecific hybridization between *V.bicolor* A and *V.negundo* B, with individuals of *V.bicolor* B and C carrying nuclear homologous sequences from both progenitors. Consequently, this led to the dispersed grouping of *V.bicolor* B and C types within the *V.negundo* B and *V.bicolor* A clades (Fig. 3). Furthermore, the plastome and nrDNA phylogenies suggest a one-way hybridization event between the maternal (*V.bicolor* A) and paternal (*V.negundo* B) progenitors. Plastid and nuclear phylogenomic discordance from hybridization events were similarly observed in *Lachemilla* (Morales‐Briones et al. 2018), *Magnolia* s.l. (Dong et al. 2022), *Piper* (Simmonds et al. 2021), and *Isodon* (Chen et al. 2022); however, genome-scale data are often needed to further validate this phenomenon. Nonetheless, this corroborated the consistent morphological differences among the *V.bicolor*OTUs established in the a priori groups. Thus, further morphometric analysis was conducted to investigate whether these observed genetic differences can be mutually elucidated using morphological markers.

### ﻿Multivariate morphometric analyses

The insubstantial evidence provided by the plastome phylogenetic trees on the species delimitation of *V.bicolor*OTUs, particularly the putative existence of interspecific hybrids (*V.bicolor* B and C), in the Philippines warranted an in-depth morphometric approach as additional evidence for their species delimitation. To achieve this, we conducted morphometric analyses using herbarium specimen measurements previously employed by Sengun (2017) for species delineation within the *V.trifolia* complex, particularly the *V.bicolor*OTUs with *V.negundo* B. This was augmented by our herbarium measurements to provide more comprehensive representation for the OTUs involved (Table 4).

**Table 4.** Number of specimens per OTU sampled for the morphological analyses.

OTUs	NUMBER OF SPECIMENS
*V.bicolor* A	12 (Sengun 2017 measurements) + 5 (this study)
*V.bicolor* B	2 (Sengun 2017) + 33 (this study)
*V.bicolor* C	9 (this study)
*V.negundo* B	2 (Sengun 2017 measurements) + 18 (this study)

**Figure 4.**
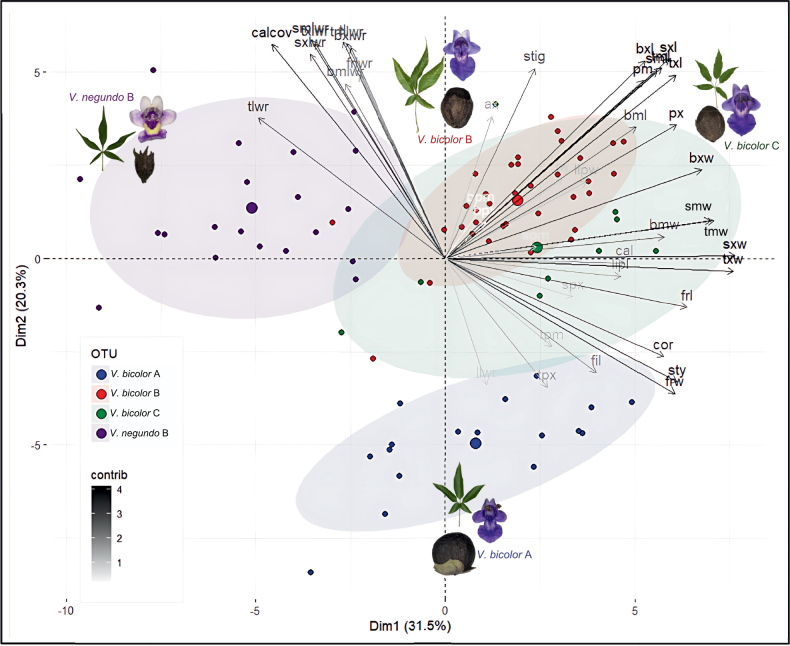
PCA biplot of *Vitexbicolor* (A–C) and *V.negundo* B accessions based on 42 quantitative characters, where the arrows represent contributions of the trait along the two principal components. Blue circles, *V.bicolor* A (N = 17); red circles, *V.bicolor* B (N = 35); green circles, *V.bicolor* C (N = 9); purple circles, *V.negundo* B (N = 20).

The first two principal components accounted for 51.8% of the total variation (Fig. 4), while 73% of the total variance could be explained by the first five components. It was observed that the first principal component primarily explained the variations in length and width measurements of the leaflets, floral parts, and fruit. Conversely, the second principal component predominantly reflects variations in the length-to-width measurements of these parts. The third principal component, contributing an additional 9.9% to the total variations, was found to be significantly influenced by variables related to petiolule lengths. These suggest that a substantial portion of the variations can be effectively explained by the first three principal components.

**Figure 5.**
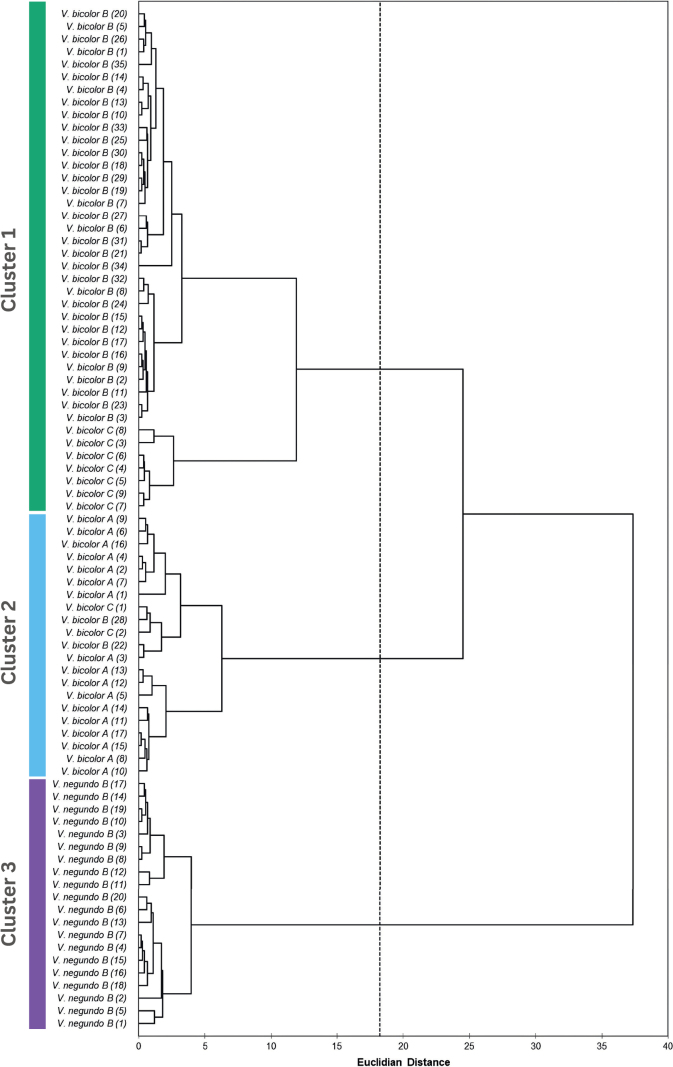
Dendrogram of the hierarchical cluster analysis (HCA) applied on the morphological traits of 81 herbarium samples. The scale on the bottom corresponds to the Euclidian distance between each class. The dendrogram is truncated to form three clusters based on entropy values.

The biplot analysis provided clear delineation among *V.bicolor* A, *V.bicolor* (B and C), and *V.negundo* B. This further supports the hypothesis that *V.bicolor* B and C belong to a distinct single taxonomic unit, separate from *V.bicolor* A and *V.negundo* B. Furthermore, these accessions can potentially be distinguished by near-orthogonal variables, particularly length-width measurements, and their ratios, including calyx coverage. The cluster analysis yielded similar insights, as the entropy-based truncation resulted in three distinct clusters. Cluster 1 comprised *V.bicolor* (B and C) accessions, cluster 2 predominantly consisted of *V.bicolor* A accessions, and cluster 3 included *V.negundo* B accessions (Fig. 5). Hence, based on the elucidated morphometric and phylogenetic data, we propose the treatment of *V.bicolor* B and C as single distinct taxonomic unit which is a potential interspecific hybrid between *V.bicolor* A (maternal) and *V.negundo* B (paternal).

To further confirm this, a linear discriminant analysis using the revised OTU classification, wherein *V.bicolor* B and C were treated as a single taxonomic unit, was performed. Remarkably, significant Fisher’s p-values were observed between the revised OTUs, and 100% correct classification was achieved in the subsequent confusion matrix using data from accessions with complete measurements (Table 5). Furthermore, analysis of Fisher’s distance from the resultant OTUs revealed insightful patterns in the morphological characteristics of the examined accessions. These suggested that *V.bicolor* B and C bore closer morphological resemblance to both *V.bicolor* A and *V.negundo* B, compared to the similarity between the latter two taxa. These findings reciprocally illuminate the hypothesis derived from the earlier phylogenetic analysis, indicating that *V.bicolor* B and C are putative interspecific hybrids and should be treated as a distinct taxonomic unit.

**Table 5.** Differences between clusters based on 42 morphological traits and their percent correct classification from discrimination analysis of 66 herbarium specimens.

**(A) Pairwise comparisons. Fisher’s p values in lower triangle, Fisher distances in upper triangle (computed in XLSTAT 2016)**						
	***V.bicolor* A**		***V.bicolor* B & C**		***V.negundo* B**	
***V.bicolor* A**	0		48.662		109.035	
***V.bicolor* B & C**	<0.0001		0		44.461	
***V.negundo* B**	<0.0001		<0.0001		0	
**(B) Discriminant analysis (computed in XLSTAT 2016)**						
**FROM\TO**	***V.bicolor* A**	***V.bicolor* B & C**		***V.negundo* B**	**TOTAL**	% **CORRECT**
***V.bicolor* A**	9	0		0	9	100.00%
***V.bicolor* B & C**	0	38		0	38	100.00%
***V.negundo* B**	0	0		19	19	100.00%
**TOTAL**	9	38		19	66	100.00%

### ﻿Univariate morphometric analyses

**Figure 6.**
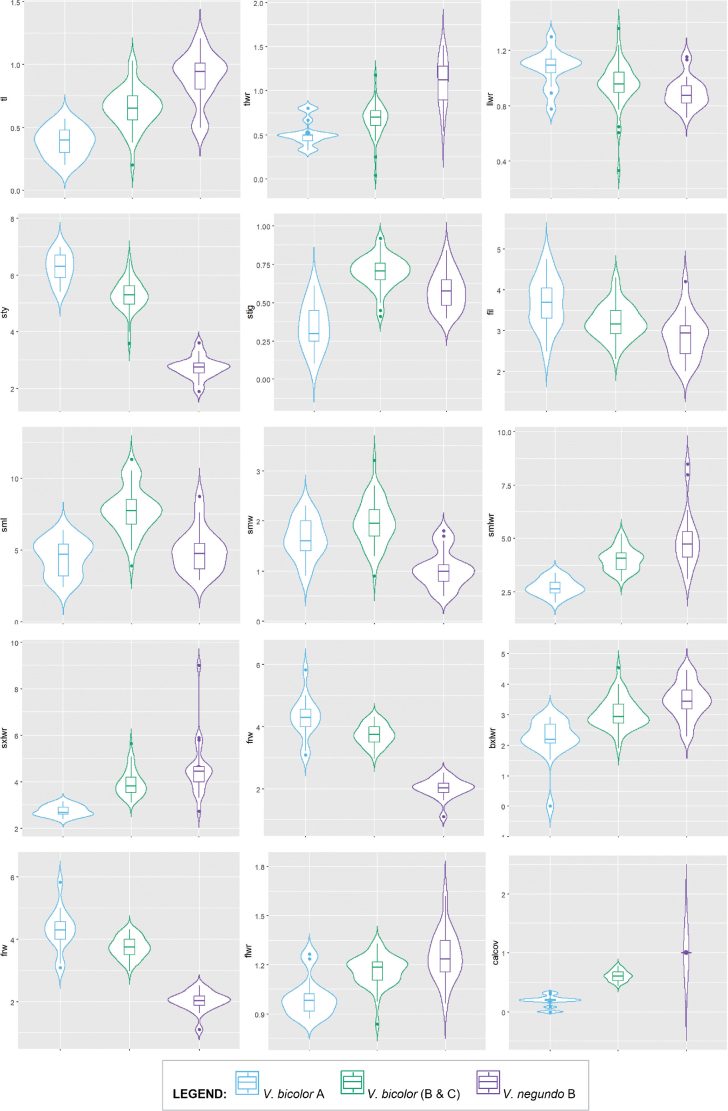
Dispersion of values for the 12 discriminating variables in the three *Vitex*OTUs.

To further identify potential characters that could delineate the resultant OTUs, univariate parametric and non-parametric tests of difference were performed. Out of the 42 characteristics examined, 38 traits exhibited significant variations among *V.bicolor* A, *V.bicolor* B and C, and *V.negundo* B accessions (Table 6). These results indicated that *V.bicolor* A had smaller, rounder leaflets, shorter petioles and inflorescence axis, larger globose fruits, and a different overall floral structure than that of *V.bicolor* B and C. Then, out of the 38 characters that were significantly varying, 12 were highly informative and exhibited significant pairwise differences across the three OTUs (Fig. 6). Most of these traits were used to construct the dichotomous key provided in the revised taxonomic treatment of the *V.trifolia* complex in the Philippines.

**Table 6.** Differences between clusters based on 42 morphological traits and their percent correct classification from discrimination analysis of 66 herbarium specimens.

TRAIT	*Vitexbicolor* A	*Vitexbicolor* B & C	*Vitexnegundo* B
**txl**	8.9±0.44^b^	12.4±0.25^a^	8.9±0.34^b^
**txw**	3.0±0.15^a^	3.1±0.06^a^	2.1±0.13^b^
**txlwr**	3.0±0.09^x^	4.1±0.06^y^	4.6±0.27^y^
**tml**	6.0±0.37^b^	10.0±0.26^a^	6.4±0.34^b^
**tmw**	2.1±0.12^a^	2.4±0.08^a^	1.5±0.10^b^
**tmlwr**	3.0±0.16^x^	4.2±0.09^y^	4.5±0.27^y^
**sxl**	7.0±0.3^b^	10.5±0.25^a^	7.3±0.28^b^
**sxw**	2.5±0.11^a^	2.7±0.07^a^	1.7±0.11^b^
**sxlwr**	2.8±0.06^x^	3.9±0.09^y^	4.6±0.29^y^
**sml**	4.5±0.30^b^	7.8±0.24^a^	4.9±0.33^b^
**smw**	1.6±0.96^b^	2.0±0.07^a^	1.0±0.08^c^
**smlwr**	2.7±0.10^x^	4.0±0.08^y^	4.9±0.31^z^
**bxl**	3.3±0.31^b^	5.6±0.19^a^	3.9±0.24^b^
**bxw**	1.4±0.15^b^	1.9±0.07^a^	1.1±0.08^b^
**bxlwr**	2.2±0.16^c^	3.0±0.08^a^	3.5±0.13^b^
**bml**	2.0±0.25^b^	3.2±0.16^a^	1.9±0.17^b^
**bmw**	0.9±0.12^a^	1.0±0.05^a^	0.6±0.07^b^
**bmlwr**	2.0±0.16^x^	3.1±0.12^y^	3.6±0.26^y^
**tpx**	1.5±0.11^y^	1.0±0.08^x^	0.8±0.08^x^
**tpm**	0.7±0.09^y^	0.5±0.08^xy^	0.3±0.05^x^
**spx**	0.9±0.10	0.8±0.09	0.6±0.08
**spm**	0.2±0.05	0.3±0.06	0.2±0.04
**bpx**	0.0	0.0	0.0
**bpm**	0.0	0.0	0.0
**px**	4.7±0.36^x^	7.0±0.22^y^	4.1±0.16^x^
**pm**	2.4±0.19^x^	4.7±0.17^y^	2.6±0.17^x^
**ax**	10.8±0.98^b^	15.1±0.58^a^	14.0±0.58^a^
**cal**	1.9±0.08^xy^	2.0±0.05^y^	1.6±0.07^x^
**tl**	0.4±0.03^c^	0.7±0.02^b^	0.9±0.05^a^
**tw**	0.8±0.07	0.9±0.03	0.8±0.03
**tlwr**	0.5±0.04^x^	0.7±0.03^y^	1.1±0.06^z^
**cor**	4.6±0.21^y^	4.4±0.04^y^	3.4±0.13^x^
**lipl**	3.0±0.20^ab^	3.2±0.08^a^	2.6±0.11^b^
**lipw**	2.8±0.17^x^	3.3±0.06^y^	3.0±0.15^xy^
**llwr**	1.1±0.04^x^	1.0±0.03^xy^	0.9±0.03^y^
**sty**	6.3±0.15^z^	5.3±0.08^y^	2.7±0.09^x^
**stig**	0.3±0.05^x^	0.7±0.02^z^	0.6±0.03^y^
**fil**	3.7±0.17^a^	3.2±0.06^b^	2.9±0.12^c^
**frl**	4.3±0.17^x^	4.3±0.06^x^	2.5±0.09^y^
**frw**	4.3±0.18^z^	3.7±0.05^y^	2.0±0.07^x^
**flwr**	1.0±0.03^c^	1.2±0.01^b^	1.3±0.03^a^
**cov**	0.2±0.03^z^	0.6±0.01^y^	1.0±0.00^x^

^a, b, c^significant differences observed using ANOVA and HSD tests; ^x, y, z^significant differences observed using Kruskal-Wallis and Dunn’s non-parametric tests.

### ﻿*V.negundo* B as *Vitexelmeri* Moldenke

Integrating plastome, nrDNA and morphological data, enough taxonomic evidence was collected to establish *V.negundo* B as a separate species, distinct from *V.negundo* A. Grouping the two OTUs will result in a paraphyletic taxon as inferred from the phylogenetic tree generated from chloroplast genome and nrDNA sequences. Throughout the multiple revisions in the history of the *Vitextrifolia* complex, specimens associated with *V.negundo* B (*Ramos* 8292, *Merril* 3627, *Darling* 16562, *McGregor* 5259) were cited Lam (1919) as *V.negundo* during the revision of Verbenaceae of the Malayan Archipelago. However, it was not until the publication of Moldenke in 1978 that this morphotype was recognized as a separate species, *V.elmeri* Moldenke. Notably, he published *V.elmeri* as a new species after he had already reviewed and revised most of the *Vitex* species in his series of publications on the “Materials toward a monograph of the genus *Vitex*” (Moldenke 1957). In the same publication, he had already examined *Elmer* 5611, the later designated holotype of *V.elmeri*, and identified it to be *V.negundo*. This clearly indicated that Moldenke, as an experienced observer and upon careful examination, was convinced that there were enough morphological differences to establish the taxon as a separate species. Moldenke (1978) identified that this species was often confused with *V.negundo*; however, he indicated that, “the aspect of its inflorescences is somewhat reminiscent of a depauperate *V.agnus-castus* L., but the pubescence is not at all mealy—canescent or albidous as in that species”. In the same publication, he also indicated that the species has only entire leaflets, contrasting with the current amended description of *V.negundo* which always has dentate leaflets (Sengun et al. 2024). Examining herbarium specimens under *V.negundo*, we observed that only specimens from the Philippines (*V.negundo* B) bear completely entire leaflets at maturity. Despite the similarity, *V.elmeri* can also be delineated by its villous indumentum observed at the veins of the abaxial leaf surface (drying with prominent veins in the leaf undersurface), brown-colored fruit at maturity, shorter style, and sparsely pubescent leaf undersurface. Thus, we propose to reinstate *V.elmeri* Moldenke as a separate species from *V.negundo*.

### ﻿*V.bicolor* B and C as new species

The pieces of taxonomic evidence support the introgression hypothesis, suggesting that *V.bicolor* B and C is a potential interspecific hybrid between proto-*V.bicolor* A and proto-*V.elmeri* (*V.negundo* B) individuals. Consequently, it should be treated as a separate new taxon, *V.arvensis* Gentallan, Sengun & M.B.Bartolome. Similar to *V.elmeri*, *V.arvensis* is often confused with *V.negundo*. Attempts to formally treat this species in the Philippines had been recorded. The earliest publication with concurring characteristics of this species dates to the publication of Blanco in 1845, under the species name *V.leucoxylon* Blanco (Blanco 1845). Although *Vitexleucoxylon* is an illegitimate name, the neotypified specimen of this species, *Merrill Sp. Blanc.* 440 (neo L [L.2768327]*), should be correctly interpreted as *V.arvensis*. Moldenke (1978) published an infraspecies taxon associated with this morphotype, V.negundovar.philippinensis Moldenke, more than 20 years after his attempt to publish a monograph of the genus *Vitex* (Moldenke 1957).

Over time, specimens from this new taxon have been usually placed in either *V.negundo* L. (Merrill 1912; Merrill 1918; Merrill 1923; Moldenke 1957; Moldenke 1978) or *V.bicolor* Willd. (Lam 1919; De Kok and Sengun 2020; Sengun et al. 2024). This is likely due to the fact that it shares characteristics, often in intermediate form, with its putative parents, *V.elmeri* (*V.negundo* B) and *V.bicolor*, resulting from interspecific hybridization.

With *V.leucoxylon* as an illegitimate name and no other species-level names published in the literature, we propose identifying *V.bicolor* B and C types as *V.arvensis*, a new endemic putative interspecific hybrid of *Vitex* in the Philippines.

### ﻿Taxonomic treatment of *V.trifolia* complex from the Philippines

#### ﻿Key 1. Key to species belonging to *Vitextrifolia* complex in the Philippines

**Table:** 

1	Leaves 1-foliolate, prostrate shrubs, rooting at the nodes	***V.rotundifolia***
–	Leaves (1–)3–5-foliolate; small trees or shrubs, never rooting at the nodes	**2**
2	Leaves (1–)3-foliolate; terminal and lateral leaflets sessile	***V.trifolia***
–	Leaves (1–)3–5-foliolate; terminal leaflet petiolulate, lateral leaflets subsessile to petiolulate	**3**
3	Leaflets always entire when young, undersurface densely pubescent drying white; cyme inflorescence unit regularly dichasially branching; fruit globose, drying black at maturity, covered by the calyx by 1/3 of its length	***V.bicolor***
–	Leaflets mostly dentate when young, occasionally entire, undersurface sparsely pubescent not drying white; cyme inflorescence unit irregularly dichasially branching; fruit ellipsoid, drying brown at maturity, covered by the calyx by more than 1/2 of its length	**4**
4	Terminal leaflet narrowly elliptic, 5.0–12.4 by 1.5–2.5 cm; inflorescence with persistent flowers in compact inflorescence units; fruit 2.0–3.1 by 1.6–2.1 mm, always fully enclosed by the calyx at maturity	***V.elmeri***
–	Terminal leaflet elliptic to lanceolate, 10.4–13.5 by 2.6–3.6 cm; inflorescence often with caducous flowers, leaving a clear set of scars on the axis, in lax inflorescence units; fruit 4.1–4.8 by 3.4–4.1 mm, not fully enclosed by the calyx at maturity	***V.arvensis***

##### ﻿1. Vitex arvensis

Taxon classificationPlantaeLamialesLamiaceae

Gentallan, Sengun & M.B.Bartolome sp. nov.

C2018846-DDF3-58CA-AC4D-14B657D4A714

urn:lsid:ipni.org:names:77350665-1

**Figure 7.**
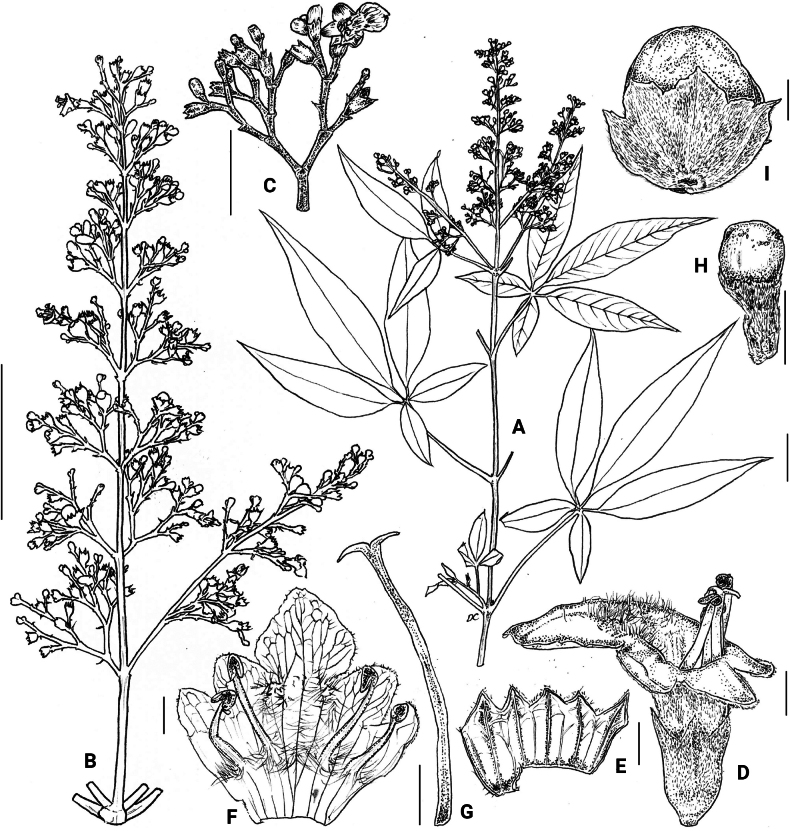
*Vitexarvensis***A** habit **B** part of inflorescence **C** side view of flower **D** calyx open, adaxial **E** Corolla open **F** style fruit in **G** calyx **H** fruit in cross-section **I** calyx. Illustration by Daryl Ceribo. Scale bars: 2.5 cm (**A, B**); 1 cm (**C**), 1 mm (**D, E, F, G, H, I**).

**Figure 8.**
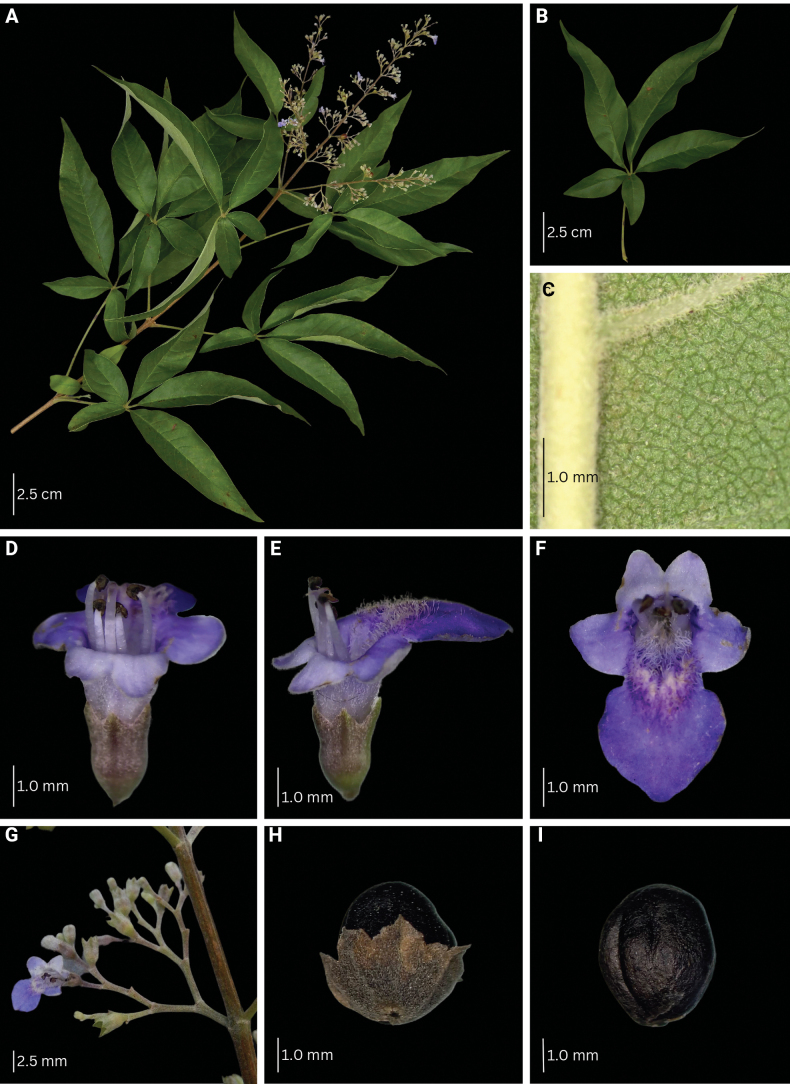
Morphological characteristics of *V.arvensis***A** branch with inflorescence **B** sixth fully unfolded leaf **C** abaxial leaf lamina surface **D–F** flower **G** inflorescence unit **H–I** mature fruit with and without calyx.

**Figure 9.**
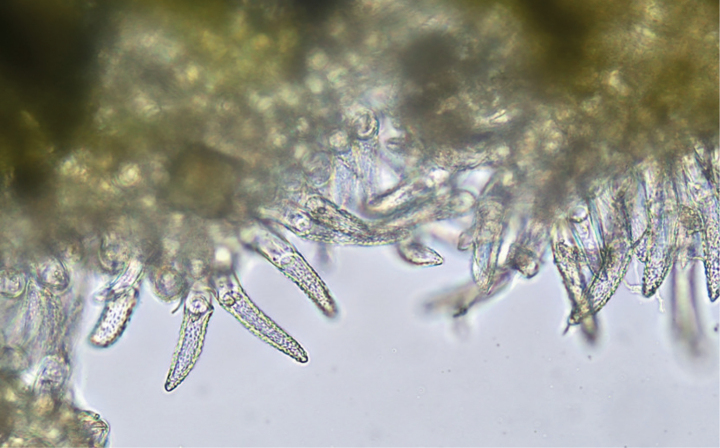
Leaf undersurface pubescence of *V.arvensis* (40× magnification). Photos: L. E. Endonela.

**Figure 10.**
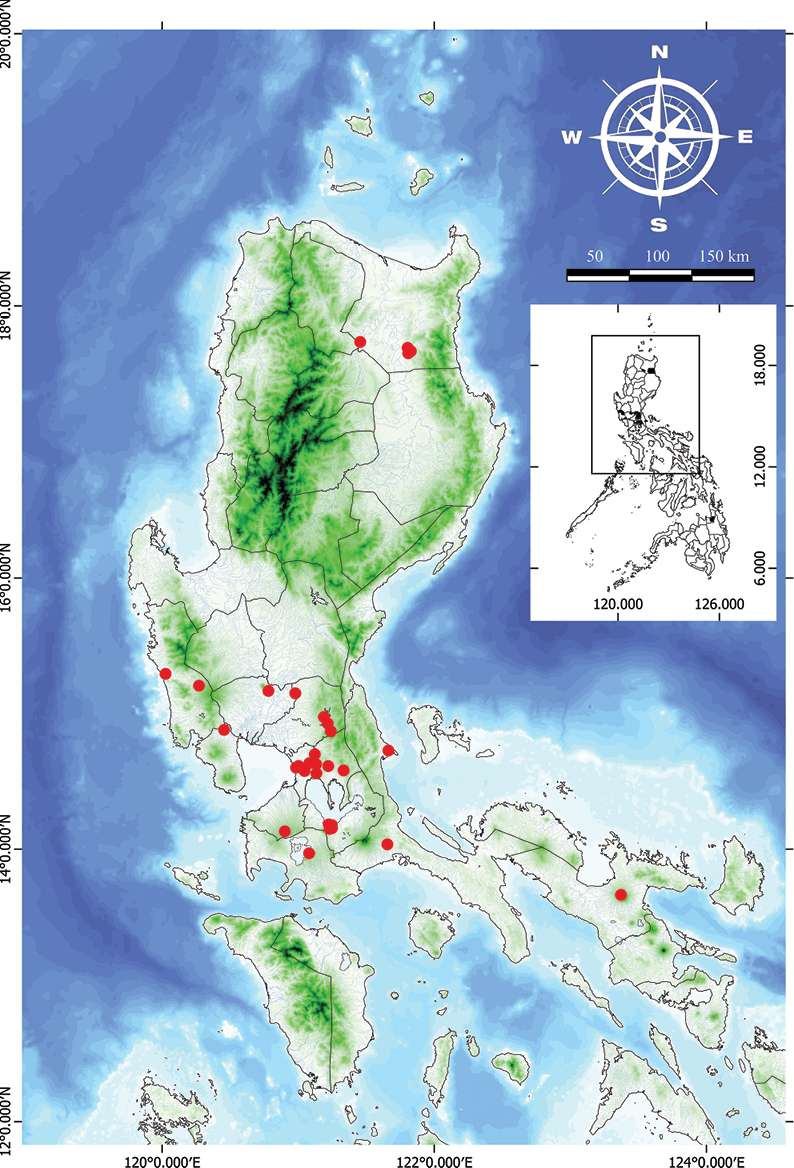
Natural distribution of *Vitexarvensis* in the Philippines.

**Figure 11.**
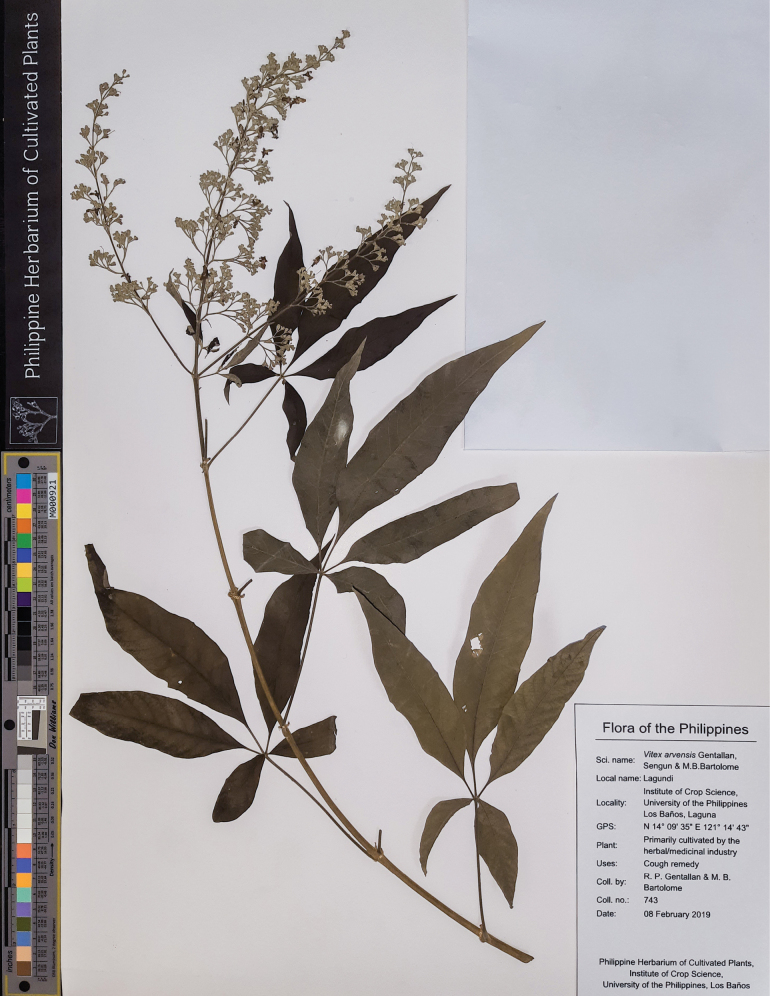
Holotype specimen of *Vitexarvensis*.

Figs 7 , 8 , 9 , 10 , 11

Vitex leucoxylon Blanco, Fl. Filip. 516. 1837. nom. illeg., not Vitexleucoxylon L.f. Type: Philippines, Rizal, Antipolo, 13 Jan. 1914, Merrill Sp. Blanc. 440 (neotype: L [L.2768327, designated by Sengun et al. (2024)]).
Vitex negundo var. philippinensis Moldenke, Phytologia 38: 308. 1978. Type: Philippines, Laguna, Los Baños, Apr., 1906, A.D.E. Elmer 8125 (holotype: PNH, destroyed; isotypes: K, NY [NY00138511]). syn. nov.

###### Diagnosis.

This new species differs from *Vitexelmeri* Moldenke by having terminal leaflet which is narrowly elliptic to lanceolate, 4.3–16.0 by 1.0–3.7 cm, 2.4–5.7 times as long as wide, and with abaxial surface moderately pubescent (vs *V.elmeri* terminal leaflet narrowly elliptic, 4.0–12.0 by 0.7–3.5 cm, 2.5–8.4 times as long as wide); inflorescence with caducous flowers, leaving a clear set of scars on the axis (vs *V.elmeri* inflorescence with persistent flowers); fruit 3.0–4.9 by 3.0–4.3 mm (vs *V.elmeri* bears 1.8–3.1 by 0.4–0.8 mm fruits fully covered by the calyx at maturity).

###### Type.

Philippines. Laguna: Los Baños, in the field genebank of the Institute of Crop Science, University of the Philippines Los Baños, 14°09′35″N, 121°14′43″E, 8 Feb. 2019, *R.P. Gentallan & M.B. Bartolome 743* (holotype: ICROPS).

###### Description.

Shrub to small tree, 2–4 m high. Leaves (1–)3–5-foliolate, reflexed to drooping; terminal leaflet elliptic to lanceolate, 4.3–16.0 by 1.0–3.7 cm, 3.0–5.0 times as long as wide, terminal petiolules 0–2.3 cm long, moderate olive green (137B) above, grayish yellow green (191A to 191B) below; secondary veins 10–17 pairs; lateral leaflets 3.9–14.5 by 0.9–3.4 cm, 2.4–5.7 times as long as wide, lateral petiolules 0–2.5 cm long, secondary veins 8–11 pairs; basal leaflets when present, 1.9–8.4 by 0.4–3.2 cm, 2.3–4.0 times as long as wide, basal petiolules 0.4–3.2 cm; apex short to long acuminate, base attenuate, acute to short acuminate, or rounded, margin entire, but occasionally dentate when young; abaxial leaf surface sparsely to moderately pubescent, hairs whitish; petiole 2.0–10.3 cm long, round to tetragonal in cross-section, puberulent. Inflorescence lax paniculate, axis 8.8–23.5 cm long, with primary branch at panicle base and along panicle axis, consisting of lateral cymes in lax clusters attached to the panicle axis or primary branch, with the oldest terminal flower often unfertilized at maturity, angular in cross-section, pubescent; bracteole triangular, up to 2 mm long, apex acute, tomentose. Calyx 5-lobed, 5-ribbed; lobes 0.2–1.3 by 0.6–1.25 mm, acute, velutinous; flowering calyx 1.0–2.49 mm long; fruiting calyx cup-shaped, 3.0–4.3 mm diameter, covering 1/2 to 3/4 of the mature fruit. Corolla 5-lobed, strong violet to light violet (N88B to N88C), outside covered with appressed hairs; lower lip broadly ovate to orbicular, 2.0–4.5 by 2.4–4.5 mm, apex acute to obtuse, margin entire, often patent, strong violet (N89B), yellowish at base, with white and yellowish hairs at corolla mouth; corolla throat inner diameter 2.05–2.53 mm; lateral lobes 1.6–2.1 by 1.6–2.0 mm, apex round to acute, often patent to reflexed, light violet (N88C); upper lip 2-lobed, lobes 1.3–1.7 by 1.4–1.9 mm, apex acute, often patent, light violet (N88D); tube infundibular 3.5–4.7 mm long, very pale purple (85D) to very light purple (85C). Stamens strongly didynamous, exserted up to 1/2 of its length, filaments 2.3–4.3 mm long, inserted 1/2 of the way on the corolla tube. Ovary 0.7–1.2 by 0.6–1.2 mm, globose, glabrous; style 3.6–6.6 mm long; stigma lobes 0.4–0.9 mm long. Fruit brown when mature; dried ellipsoid to ovoid, 3.0–4.9 by 3.0–4.3 mm, apex notched to truncated, glabrous with sparse glands.

###### Phenology.

Produces fruits and flowers all year round.

###### Habitat and ecology.

Growing primarily in farmlands, home gardens under cultivation as medicinal and/or hedge article, but recorded in secondary forests, mixed thickets, along rivers, trails and ridges, open places and in wastelands. Altitude: 15–500 m.

###### Distribution.

Endemic in the Philippines, distributed primarily from Central to South Luzon but widespread throughout in cultivation.

###### Vernacular names.

lagundi (Tagalog, Bisaya), dangla (Ilokano, Zambales)

###### Conservation status.

IUCN assessment gives the extent of occurrence (EOO) and area of occurrence (AOO), as greater than the threshold for the vulnerable category, thus the status would be least concern (LC).

###### Notes.

1. This taxon has never been recognized at the species level before. The specific epithet *arvensis* provides insight into its usually cultivated nature since it is primarily cultivated on a commercial scale, and in home gardens as a medicinal plant.

2. This is a putative natural interspecific hybrid between *V.bicolor* and *V.elmeri*. Although now distributed across the Philippines, it was primarily distributed in areas where *V.elmeri* and *V.bicolor* overlap, from South to Central Luzon. This species bears resemblance to both parents: *V.elmeri* in terms of the yellow corolla ridge color, presence of villous abaxial secondary veins, sparse pubescence on the lamina undersurface, and having dentate leaves when young; and *V.bicolor* in terms of lax inflorescence units, flower color, and leaf size.

3. Aside from bearing caducous flowers, this new species further differs from *Vitexbicolor* Willd. by having the mature ovoid fruits covered by the calyx by 1/2–3/4 along its length which dries brown, while *V.bicolor* has larger (5.2–5.8 by 4.3–5.6 mm) globose fruits covered by the calyx to only up to 1/5 along its length which dries black.

4. Unlike *V.bicolor*, *V.arvensis* was observed to have a lower germination rate of seeds, with virtually non-existent seedlings under the canopy. However, with interventions, these can be germinated ex-situ, albeit with a low success rate.

###### Documented uses (in the Philippines).

Leaves heated and applied as external patch or as liniment for fever, fruits and leaves used as an insecticide; juice extracted from leaves by grinding ingested for cough relief; decoction or boiling of leaves for fever and cough.

###### Specimens examined.

**Philippines. Cagayan**: Peñablanca, San Roque, May 1917, Adduru 173 (US03805856); Vendivil & Fernando 125366 (PNH); Peñablanca, Callao, Apr 1915, Castillo 22747 (US03805843); Tuao, May 1979, Sr. M. Rocero 165079 (PNH); **Zambales**: Botolan, May 1903, Merrill 2917 (US03805807); Botolan, Villar, Nov 1947, R. B. Fox 4928 (PNH); **Bataan**: Dinalupihan, Feb 1903, Merrill 1503 (US03805792); **Pampanga**: Arayat, Vidal 1648 (L.2768325); **Bulacan**: San Miguel, 15–20 m alt., B. Fegan 113564 (PNH); Doña Remedios Trinidad, Kabayanan, Dec 1994, H. Garcia et al. 15334 (L.4212458); Norzagaray, San Lorenzo, Mar 1996, E. Barbon & H. Garcia 22403 (L.4216246); **Manila**: Manila, Oct 1903, Merrill 3429 (US03805814); Sep 1910, M. Ramos (E01121257); near Manila, Capt. Wilkes s.n. (US 00830006); Sampaloc, Balic-balic, Aug 1890, Loher 4433 (US03805840); Mandaluyong, Malamig, G. Edaño 3783 (PNH); **Cavite**: Mendez, Mangubat 1343 (US03805791); **Laguna**: Los Baños, Dec 1903, Hallier 4055b (L.2775187); Apr 1906, Elmer 8125 (E01121256); Sep 1909, Rosenbluth & Tamesis (L.2768326); 250 m alt., Jan 1911, Holman 99 (US03805805, US03805808); Jul 1917, Elmer 18119 (US03805788, L.2768218); Makiling National Park, 100 m alt., Oct 1945, M. D. Sulit 8311 (PNH); Mar 1963, D. R. Mendoza 92452 (PNH); San Antonio, Mar 1948, E. Canicosa 9707 (PNH); College, 50 m alt., Sep 1954. J. V. Pancho 32919 (PNH); **Batangas**: Mataas na Kahoy, Arañez 14 (L.2768323); **Rizal**: Marikina, Barangka, G. Edaño 36210 (PNH); Antipolo, 100 m alt., Mar 1903, Merrill 1636 (US03805812); Jan 1913 Merr. Sp. Blanc. 440 (L.2768327); Antipolo, Boso-boso, Loher 4432 (US03805810); San Mateo, May 1904, Ahern’s collector 102 (US03805809); Taytay, Feb 1953, Lorena 18 (L.0248373); Tanay, May 1903, Merrill 2320 (US03805790); **Quezon**: Tayabas, May 1916, Cailipan 25637 (US03805789); Infanta, Sep 1904, Whitford 853 (US03805784); **Camarines Sur**: Pasacao, Dalupaon, 1901, Ahern 255 (BO1616593, US03805779), Naga City, Mt. Isarog, Oct 1992, Barbon et al. 8377 (L.3930320); **Agusan Norte**: Butuan City, Tungao, May 1991, Barbon et al. 1917 (L.4212463).

##### ﻿2. Vitex bicolor

Taxon classificationPlantaeLamialesLamiaceae

Willd., Enum. Pl. 2:660. 1809

2EC1E709-BE9B-5546-8CFB-4C2298C8ECE5

Figs 12 , 13

###### Type.

‘Habitat in India Orientale’, cult. Hort. Berlin from seeds sent by Klein (‘Ind. 1797’), Herb. Willdenow 11709 (holotype: B-W [B11709-010])

###### Description.

Shrub to small tree, 1–5 m high. Leaves (1–)–3–(5)-foliolate; terminal leaflet elliptic to narrowly elliptic, 8.9–11.6 by 3.0–3.5 cm, 2.7–3.5 times as long as wide, petiolules 0.8–1.4 cm long, secondary veins 14–18 pairs, moderate olive green above (N137A), light gray (N191C) to grayish yellow green (N191) below; lateral leaflets 5.4–9.6 by 2.5–3.7 cm, 2.1–3.3 times as wide, petiolules 0.5–0.9 cm long, secondary veins 10–14 pairs; basal leaflets when present, 2.4–4.2 by 1.5–2.2 cm, 1.1–2.5 times as long as wide; basal petiolules absent; apex acute to acuminate, base acute to cuneate, margin entire; abaxial leaf surface highly pubescent, with tomentose white hairs; petiole 3.2–4.8 cm long, round in cross-section, covered with minute hairs. Inflorescence paniculate, axis c. 17.5 cm long, with primary branch at panicle base, consisting of lateral dichasial cymes in lax clusters attached to the panicle axis or basal primary branch, with the oldest central flower often fertilized at maturity, angular in cross-section, pubescent; bracteole triangular, c. 0.75 mm long. Calyx 5-lobed, 5-ribbed; lobes 0.2–0.6 by 0.6–1.0 mm, acute, velutinous; flowering calyx 1.5–1.7 mm diameter, fruiting calyx 2.5–3.6 mm diameter, covering up to 1/5 of the mature fruit. Corolla 5-lobed, strong violet to light violet (N88B to N88C), outside covered with appressed hairs; lower lip broadly ovate to deltoid, 2.7–4.3 by 2.5–3.5 mm, apex acute, margin entire, often reflexed to patent, strong violet (N89B), purple and white at base, with white and purple hairs at corolla mouth; corolla throat inner diameter 1.7–2.4 mm; lateral lobes 1.2–1.7 by 1.2–1.9 mm, apex round, often reflexed to patent; upper lip 2-lobed, lobes 1.0–1.8 by 1.0–1.6 mm, apex rounded, often reflexed to patent, light violet (N88C); tube infundibular 5.0–6.0 mm long, light violet (N88C to N88D). Stamens strongly didynamous, shorter filaments 2.9–3.8 mm long, longer filaments 3.4–4.4 mm long, inserted c. 1/3 of the way on the corolla tube; anther 0.7–0.9 mm long. Ovary globose, glabrous; style 6.2–7.8 mm long; stigma lobes 0.3–0.6 mm long. Fruit brown to black when mature; dried ellipsoid to spherical, 5.2–5.8 by 4.3–5.6 mm, apex rounded, glabrous.

**Figure 12.**
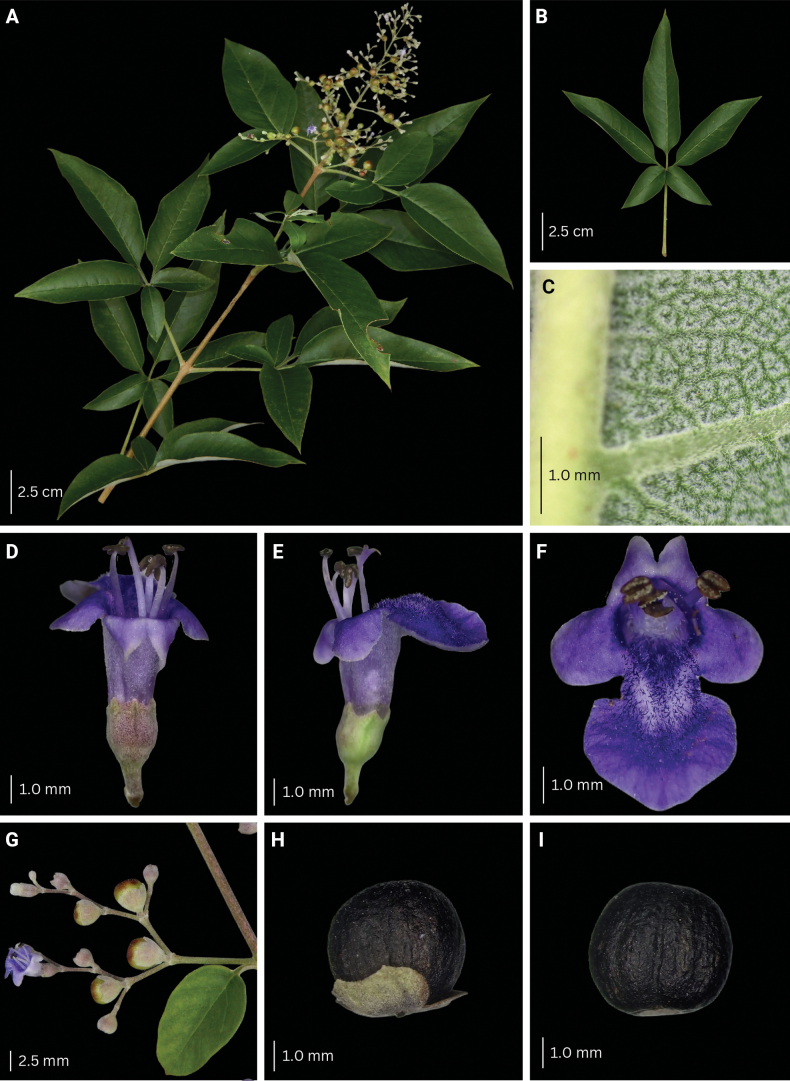
Morphological characteristics of *V.bicolor***A** branch with inflorescence **B** sixth fully unfolded leaf **C** abaxial leaf lamina surface **D–F** flower **G** inflorescence unit **H–I** immature fruit with and without calyx.

###### Habitat and ecology.

Along the seashore in beach, mangrove and littoral forests to hillsides along mountain slopes; also recorded in cultivation as hedge/fence and medicinal plant. Altitude: usually at sea level but extends up to 300 m.

**Figure 13.**
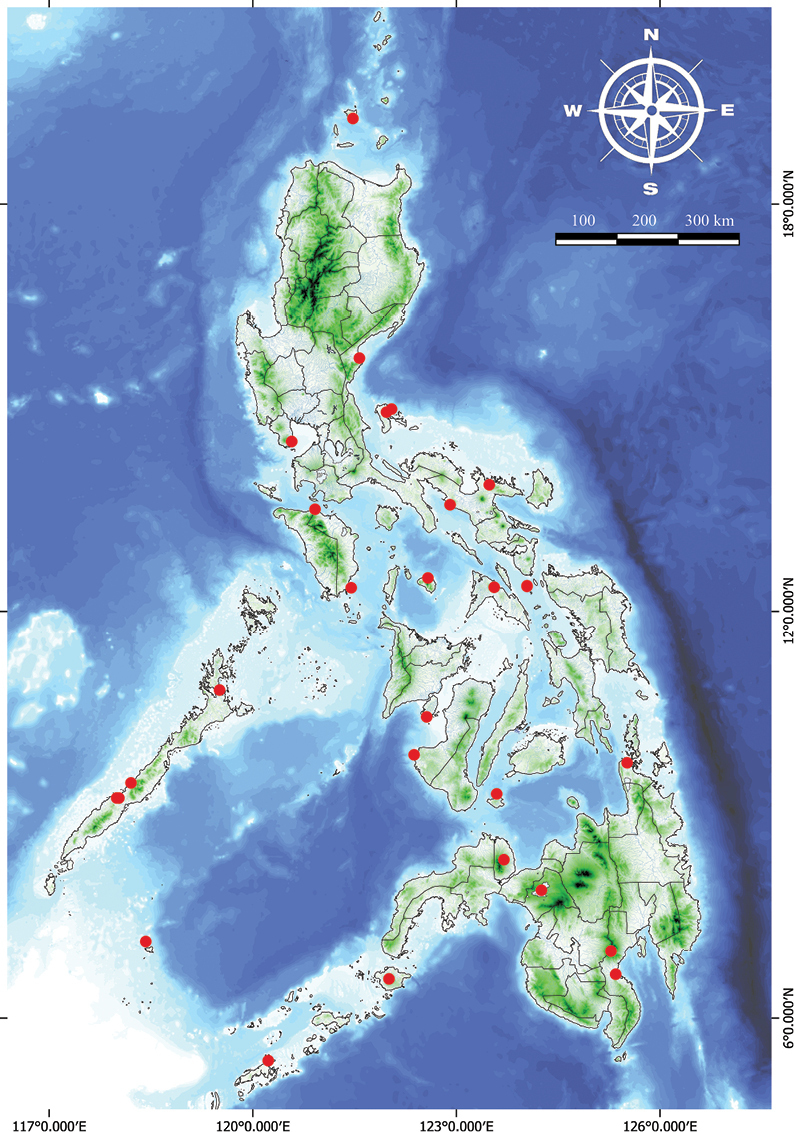
Natural distribution of *Vitexbicolor* in the Philippines.

###### Distribution.

Sri Lanka, South China: Hainan, Japan: Okinawa; Malesia: throughout, Australia: Queensland, Pacific: Tonga, Samoa and Marianas Islands. Indigenous throughout the Philippines.

###### Vernacular names.

lagundi (Tagalog, Bikolano, Bisaya), tulasi (Tagbanua, Palawan), pirabon (Palawan)

###### Notes.

This species is seldom utilized for its medicinal use in the Philippines.

###### Documented uses (in the Philippines).

Leaves for treating dengue, cough, fever; leaves applied as poultice against the forehead and/or temples as a febrifuge.

###### Specimens examined.

**Philippines. Cagayan**: Calayan, May 1961, E. Quisumbing & R. Del Rosario 79790 (PNH); **Bataan**: Limay, Lamao river, Nov 1903, R. S. Williams 185 (US03805765); Sep 1904, T. Borden 2035 (US03805781); Sep-Dec 1904, R. Meyer 2276 (US03805783, BO); **Aurora**: Baler, Sep 1902, Merrill 1106 (US03805785); **Quezon**: Burdeos, Karlagan, R. B. Fox 8991 (PNH); Botonan, Barbon et al. 2047 (L.4216303); **Oriental Mindoro**: Puerto Galera, Minolo, Apr 1952, J. V. Santos 5258 (US03805782, L.2768221); **Romblon**: Magdiwang, Silum, May 1994, Reynoso 14138 (L.4216134); **Palawan**: Taytay, 30 May 1913, Merrill Sp. Blanc. 302 (US00699536, L.2768328); Quezon, Ampaplot, Apr 1964, R. A. Espiritu 91477 (L.2768220); Tabon, Nov 1963, E. J. Reynoso 87701 (PNH); **Camarines Norte**: Lagonoy, Commerson s.n. (P00657557); **Camarines Sur**: Pasacao, Dalupaon, Apr 1901, Ahern 166 (BO); 1902, Ahern 814 (US03805804); May 1902, Ahern 223 (US03805780, BO); **Masbate**: 1904, Clark 2527 (US01962875, BO); **Guimaras**: Nueva Valencia, Igdarapdap, June 1955, A. T. Taleon 34030 (PNH); **Antique**: Caluya, Kabilo, 300 m alt., Feb 1997, Romero 29662 (L.4216249); **Negros Occidental**: Cauayan, Bulata, 50 m alt., Aug 1995, Madulid & Majaducon, 36097; **Siquijor**: Larena, Sandugan, 5–10 m alt., Oct 1998, Christenhusz 86 (U.1762597); **Samar**: San Vicente, Medio Island, Mar 1957, Y. Kondo & G. Edaño 38739 (L.2768219, PNH); **Lanao del Norte**: Aug 1926, F. Guerrero 30388 (E01121263); **Misamis Oriental**: Jan-Feb 1913, D. P. Miranda 17976 (US03805770); **Davao del Sur**: Davao City, Santa Cruz, Feb 1904, Copeland 691 (US03805774); Sep 1909, Elmer 11999 (US03805774, US03805769, L.2768222, BO, PNH); Padala, Jun 1905, R. S. Williams 2978 (US03805777); **Basilan**: Jun 1911, Tarrosa 19553 (L.2768223); **Tawi-tawi**: Mapun, Pamelican, Feb 1957, Y. Kondo & G. Edaño 39032 (PNH); Languyan, Birad-dali, May 1992, F. J. M. Gaerlan & E. C. Sagcal 10053 (L.4216299).

##### ﻿3. Vitex elmeri

Taxon classificationPlantaeLamialesLamiaceae

Moldenke, Phytologia 38: 307 (1978)

6E908D2F-9C4E-5170-855C-DB823C6F8239

Figs 14 , 15

###### Type.

Philippines, Union Province, Bauang, Feb. 1904, Elmer 5611 (holotype: NY [NY00138505]).

###### Description.

Shrub to small tree, sometimes bushy, 1–4 m high. Leaves (3–)5–7-foliolate; terminal leaflet narrowly elliptic to elliptic, 4.0–12.0 by 0.7–3.5 cm, 2.5–8.4 times as long as wide; lateral leaflets 2.9–10.0 by 0.5–2.7 cm, 2.5–9.0 times as long as wide; basal leaflets 0.6–6.7 by 0.2–1.8 cm, 1.1–4.5 times as long as wide; apex acuminate, base acute, margin entire at maturity (with dentation in the middle only when young), glabrous above to very sparsely pubescent below, with the midrib and veins covered with erect villous hairs; petiole 1.4–5.5 cm long, angular in cross-section, covered with villous hairs; petiolules 0–1.4 cm long. Inflorescences paniculate, terminal axis 3–15 cm long, angular in cross-section; flowers in dense lateral clusters on peduncles up to 5 mm long; tomentose, hairs erect; bracteoles linear up to 3 mm long. Calyx 5-lobed, clearly 5-ribbed; lobes 0.5–1.3 by 0.6–1.2 mm, clearly developed, acute to acuminate, persistent, villous; glands few; flowering calyx, excluding the lobes, 1.4–2.2 mm long; fruiting calyx 1.6–2.5 mm diameter, fully enclosing the mature fruit. Corolla 5-lobed, pink; covered with appressed hairs, glands few; lower lip 2.0–3.5 by 2.0–4.0 mm, usually reflexed, apex cuneate to acute, margin entire, two well-developed ridges at corolla mouth, yellow; lateral lobes 1.2–2.4 by 1.0–2.5 mm, apex rounded to subacute, patent. Stamens: filaments 2.1–4.2 mm long, slightly to strongly didynamous, inserted halfway on the corolla tube. Ovary globose, 0.6–1.0 by 0.6–0.9 mm, glabrous; style 2.0–3.6 mm long; stigma lobes 0.4–0.8 mm long. Fruit light to dark brown when mature; dried obovoid to cylindrical, 1.8–3.1 by 0.4–0.8 mm, apex truncate, glabrous, smooth.

**Figure 14.**
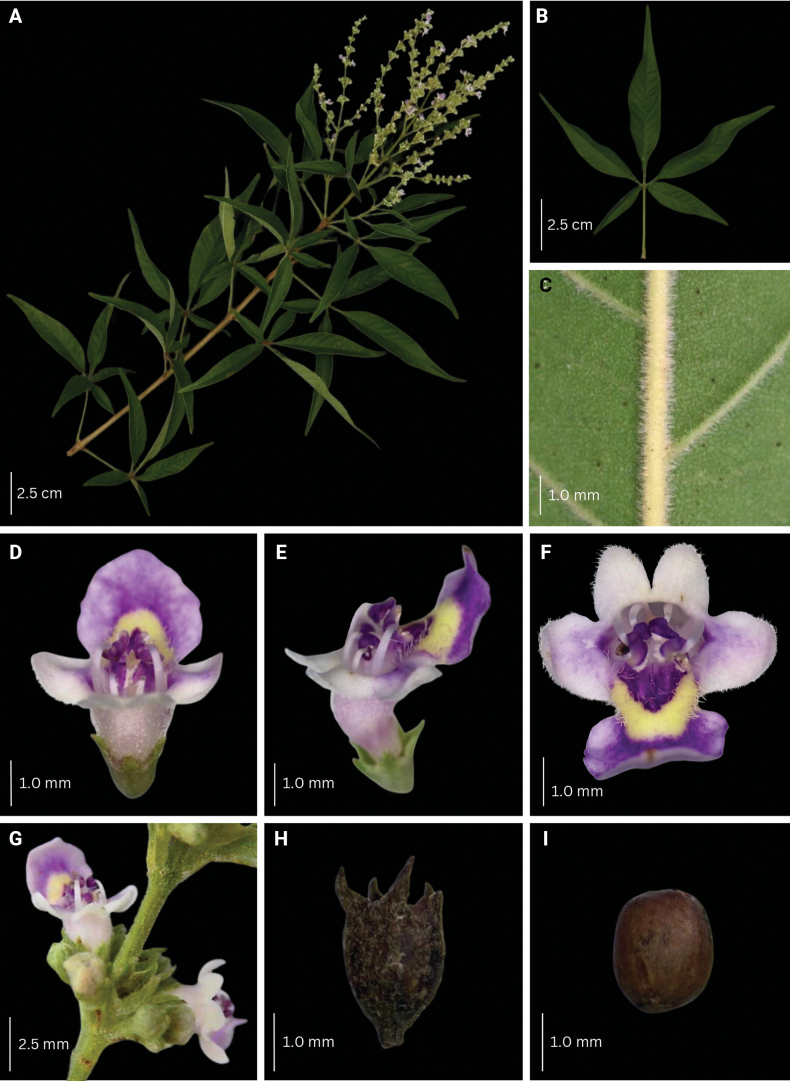
Morphological characteristics of *V.elmeri***A** branch with inflorescence **B** sixth fully unfolded leaf **C** abaxial leaf lamina surface **D–F** flower **G** inflorescence unit **H–I** mature fruit with and without calyx.

###### Phenology.

Produces flowers and fruits all year round.

###### Habitat and ecology.

Growing primarily in disturbed habitats (i.e. farmlands, cemeteries, wastelands) and in cultivation, but also recorded along thickets in secondary forests, and roadsides. Altitude: usually on hills and slopes, 50–500 m.

**Figure 15.**
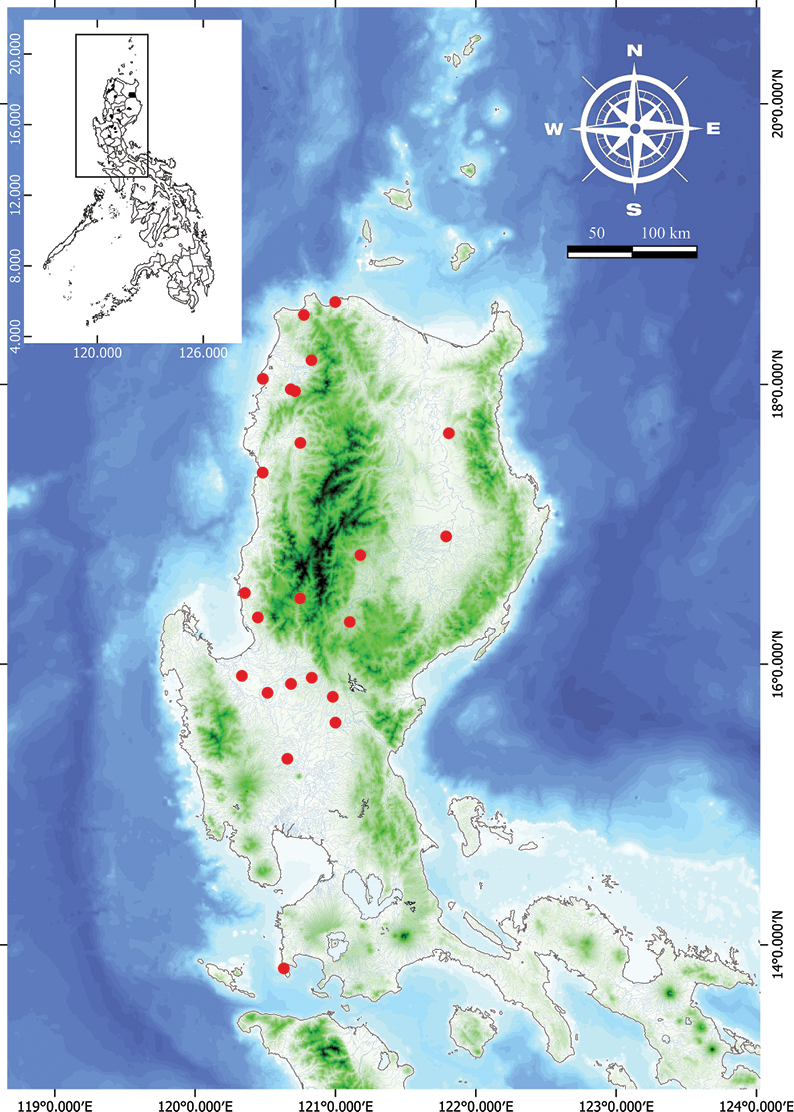
Natural distribution of *Vitexelmeri* in the Philippines.

###### Distribution.

Endemic in the Philippines, throughout North- to South-western Luzon, with cultivation records in Agusan del Norte, Iloilo, Antique.

###### Vernacular names.

lagundi (Tagalog, Bisaya), dangla (Ilokano)

###### Conservation status.

IUCN assessment gives the extent of occurrence (EOO) and area of occurrence (AOO), as greater than the threshold for the vulnerable category, thus the status would be least concern (LC).

###### Notes.

1. *V.elmeri* is currently synonymized under *V.negundo*, however, based on our integrative evidence, we propose to reinstate this taxon.

2. This species is predominantly used as a medicinal plant in the Northwestern part of Luzon. It pervades in disturbed areas, and the remaining populations may be remnants of its natural habitat which had now been developed. Their seeds also germinate in situ.

3. The species differs from *V.negundo* by having completely entire leaflets, with veins at the undersurface covered by villous indumentum, at maturity; flowers with pink lower lip; and fruits always drying brown at maturity.

4. There is a diversity of chlorotypes present for this taxon.

###### Documented uses (in the Philippines).

Decoction of leaves prepared for flu, cough, and malaria; extracted juice from leaves by grinding for cough (internal use); utilized in preference in some parts of the Ilocos region.

###### Specimens examined.

**Philippines. Ilocos Norte**: Banna, Balioeg, 100 m alt., Nov 1975, Iwatsuki 11 (L.2775183); Currimao, Bimmanga, 1984, Vendivil & Fernando 160201 (PNH); Carasi, Sinaligan, 1984, Vendivil & Fernando 160467 (PNH); Nueva Era, Sto. Niño, Nov 1986, Vendivil & Fernando 165304 (PNH); Bangui, Mt. Napidad, 500 m alt., Feb 1997, H. Garcia & L. Fernando 25321 (L.4216269); **Ilocos Sur**: Sta. Maria, Dec 1958, M. L. Steiner 39796 (PNH); **La Union**: Tubao, Anduyan, <500 m alt., Feb 1912, Vanoverbergh & Skent s.n. (BR0000026253473V); **Pangasinan**: Bautista, Jul 1903, Merrill 2876 (US03805806); Balungao, Mt. Balungao, 1908, Darling & Merritt 14051 (US03805859); Darling & Merritt 14063 (BR0000034903858); San Juan, San Carlos, May 1909, M. Ramos 8292 (L.2775179, L.2775180); Umingan, Apr 1914, Otanes 17995 (US03805811); **Mt. Province**: Ambuklao, Agno River, Nov 1953, Quisumbing 18826 (US03805838, L.2775182); Ifugao, Luta, 500 m alt., Klock 91 (US02782038); **Abra**: Feb 1909, Darling 16562 (L.27751881); M. Ramos 7215 (BR0000034903865); **Cagayan**: Sta. Praxedes, San Juan, 520 m alt., Aug 1995, H. Garcia et al. 18454 (L.4216254); Peñablanca, San Roque, 1975, Vendivil & Fernando 125368 (PNH); **Isabela**: Cauayan, Jun 1902, Merrill 147 (US03805855); **Nueva Vizcaya**: Dupax, Mar 1912, R. C. McGregor 11471 (E01121242); **Tarlac**: Concepcion, Nov 1903, Merrill 3627 (L.2775186); **Nueva Ecija**: General Mamerto Natividad, Pulong Singkamas, Sep 1908, R. C. McGregor 5259 (L.2775185); San Jose, Camanacsacan, Feb 1955, R. Martin 35234 (PNH); **Batangas**: Calatagan, 50 m alt., Jan 1949, E. Quisumbing 6578 (PNH).

##### ﻿4. Vitex rotundifolia

Taxon classificationPlantaeLamialesLamiaceae

L.f., Suppl. Pl.: 294. 1782

E35DF726-D793-5F8F-9AFF-6B6A46F16BE2

Figs 16 , 17

Vitex trifolia var. simplicifolia Cham., Linnaea 7: 107. 1832). Type: Philippines, Luzon, Cavite, Dec. 1817 – Jan. 1818, Chamisso s.n. (holotype: LE n. v.).
Vitex repens Blanco, Fl. Filip. 513. 1837. Type: Philippines, Luzon, Batangas, 11 Feb. 1915, Merr. Sp. Blanc. 814 (neotype: K [K000182650], designated by Sengun et al. (2024)).

###### Type.

Japan, Thunb. s.n. (Hb. Thunb. 14619) (holotype: LINN-SM; isotype: UPS-THUNB [V-125606]).

###### Description.

Prostrate, decumbent, or scandent shrub, usually below 1 m tall, sometimes creeping and spreading on the ground up to 4–5 m, rooting at the nodes. Leaves 1-foliolate, round to obovate-spathulate, 0.9–5.1 by 0.5–3.7 cm, 1.3–2.8 as long as wide, base cuneate, apex rounded to subacute, margin entire; upper surface moderate yellow green (147B), pubescent, especially at the midrib; lower surface grayish yellow green (191B), velutinous, covered with whitish hairs obscuring the venation and areolation; secondary veins 4–7 pairs, slightly prominent on both surfaces; petioles, when present, up to 13 mm long. Inflorescence terminal, paniculate, 5.9–11.4 cm long axis, consisting of short dichasial cymes, often unbranched, in dense clusters, with the oldest central floret usually fertilized at maturity. Calyx 5-lobed, velutinous, with appressed hairs, lobes 0.35–0.70 by 0.9–1.9 mm; flowering calyx 1.8–4.0 mm long, 2.2–3.6 mm diameter; fruiting calyx 4.1–5.4 mm diameter, covering up to 90% of the fruit at maturity. Corolla 5-lobed, pale purple, outside covered with appressed hairs; lower lip orbicular to broadly ovate, 3.0–6.10 by 2.9–6.5 mm, apex subacute, margin entire, two clear ridges at corolla mouth, often reflexed, deep violet (N89D) to strong violet (N90A), covered with white hairs at corolla mouth; tube 4.8–9.7 mm long, infundibular, light violet (N91A to N1B). Stamens with filaments 5.2–7.5 mm long, slightly didynamous, inserted 1/3 of the way on the corolla tube, exceeding the corolla mouth from 1/3 to 1/2 of its length, anthers c. 2 mm long. Ovary 1–1.5 mm diameter, globose, glabrous, the upper half covered with glands; styles 6.0–12.4 mm long, stigma 2-lobed, lobes 0.3–1.5 mm long. Fruit when dried, black at maturity, globose, 3.7–5.5 by 4.1–5.4 mm.

**Figure 16.**
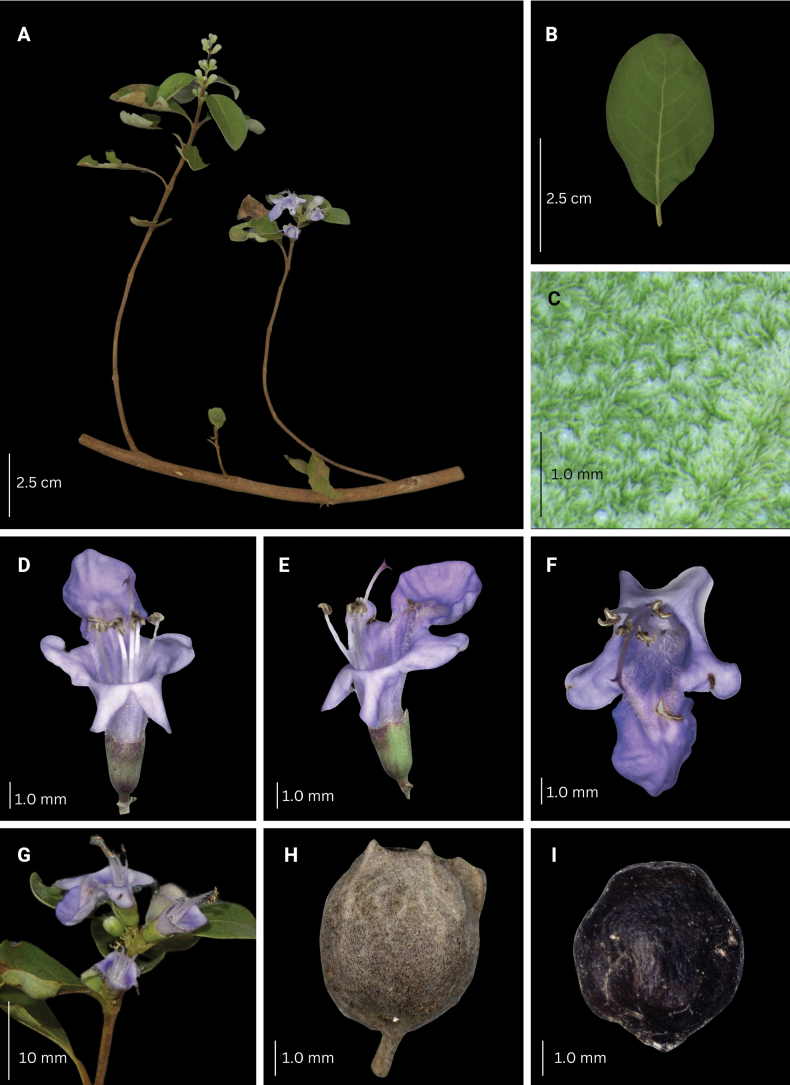
Morphological characteristics of *V.rotundifolia***A** branch with inflorescence **B** sixth fully unfolded leaf **C** abaxial leaf lamina surface **D–F** flower **G** inflorescence unit **H–I** mature fruit with and without calyx.

###### Habitat and ecology.

Usually along coastal strands and seashores, on sandy beaches along *with Ipomoea pes-caprae* and forming dense mats below the thickets of *Vitextrifolia* in the upper beach, also on sand dunes, rocky beaches, and beach forest.

###### Phenology.

Flowering and fruiting all year round.

###### Distribution.

Widespread from Japan, Korea, and eastern coast of China to north and northeast coast of Australia and extending to the Pacific until Samoa and Fiji, absent from the central Pacific with only an isolated population in Hawaii. Indigenous all throughout the Philippines, particularly toward the island borders.

**Figure 17.**
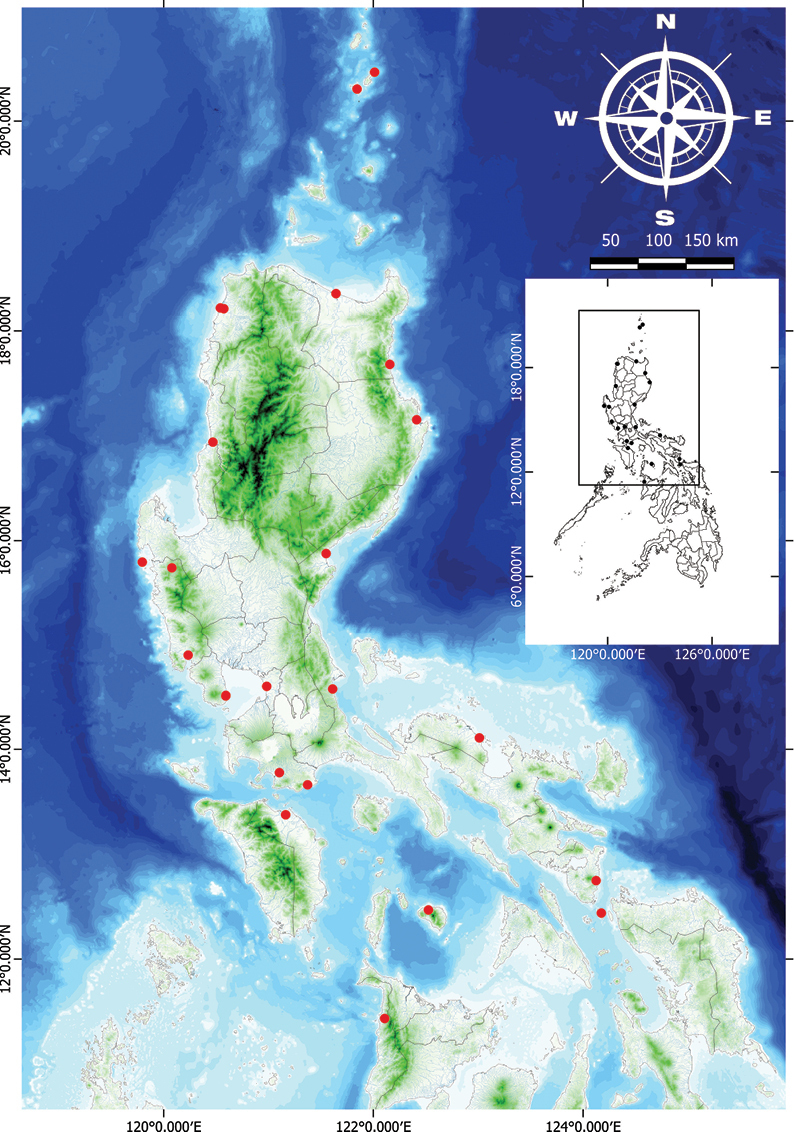
Natural distribution of *Vitexrotundifolia* in the Philippines.

###### Vernacular names.

lagunding-dagat (Tagalog), lagunding-gapang (Tagalog), dungla (Tagalog), daldalaki (Ilokano), dangla-ti-baybai (Ilokano), bantige (Ilokano), agubarau (Bisaya).

###### Notes.

In Sengun at al. (2024), p. 115, *Merrill Species Blancoanae 814* has been mistakenly identified as *V.trifolia*. It is indeed *V.rotundifolia*.

###### Documented uses (in the Philippines).

This species is rarely used for its medicinal properties, with the leaves being boiled or used in decoctions to treat cough.

###### Specimens examined.

**Philippines. Batanes**: Sabtang, 26 Mar 1961, E. Quisumbing et al. 79299 (PNH); Batan, Mt. Iraya, 20 Mar 1991, Barbon et al. 1581 (L.4212461); **Ilocos Norte**: Laoag, Sta. Maria, Apr 1955, J. V. Santos 6267 (US03805794); Laoag, 3 Jan 1965, Br. Alfred 93787 (PNH); **Ilocos Sur**: Tagudin, Aug 1909, R. C. McGregor 10072 (BR0000034904954); **Cagayan**: Aparri, June 1902, E. D. Merrill 323 (US03805761); Peñablanca, Baguio cove, 14 Apr 1981, M. S. Allen 150236 (L.2768427); **Isabela**: Palanan, Palanan bay, June 1913, L. Escritor 21171 (US03805797); **Bataan**: Limay, Lamao river, Sep 1904, T. E. Borden 1940 (US03805793, E01121261); Lamao, 1907, H. M. Curran s.n. (BO1621593); **Zambales**: Subic, Sep 1903, H. Hallier 4230 (L.2768419, L.2768425); Sta. Cruz, Hermana Mayor Island, Dec 1954, J. V. Santos 6210 (L.2768426, US03805794); Sta. Cruz, Acoje Mining, Aug 1954, J. Yench 18928 (PNH); **Central Luzon**: Loher 4434 (US03805799); **Manila**: 1914, Perrottet s.n. (L.2768423); **Batangas**: Batangas City, San Pedro, Feb 1915, Merrill Sp. Blanc. 814 (US00623757, BO1359175); San Juan, Hugom, Oct 1946, M. D. Sulit 7440 (BR0000034904947); **Aurora**: Dipaculao, Mijares (Gupa beach), May 1992, Stone et al. 5475 (L.4212443); **Quezon**: Infanta, Sep 1904, H. N. Whitford 755 (US03805755); **Oriental Mindoro**: Calapan, Apr 1903, E. D. Merrill 898 (US03805798); June 1906, L. Mangubat 926 (US03805759); **Romblon**: Magallanes, Mt. Giting-giting, Apr 1910, A. D. E. Elmer 12135 (L.2768429, U.1762519, US03805796, E01121262, BO1621591); **Camarines Norte**: Daet, Marcedes, 1903, H. Hallier 4230a (L.2768421, L.2768420); **Sorsogon**: Bulusan, near town, June 1958, J. Sinclair & G. Edaño 9673 (L.2768422, US03805795; E01121264); **Panay**: Antique, Culasi, May-Aug 1918, R. C. McGregor 32477 (L.2768418, BO1359176); **Samar**: Capul, 27 Mar 1957, Y. Kondo & G. Edaño 36800 (PNH).

##### ﻿5. Vitex trifolia

Taxon classificationPlantaeLamialesLamiaceae

L., Sp. Pl.: 638, 938. 1753

B7007935-6676-588E-9D8D-8AB201B6C490

Figs 18 , 19

###### Type.

India, Herb. Linn. 811/7 (holotype: LINN)

###### Description.

Shrub, prostrate to erect, to small tree, up to 3 m high. Leaves (1–)3-foliolate; elliptic to obovate or obovate-spatulate, grayish olive green (NN137A) to moderate olive green (N137A) above, light greenish gray (N191C) to light gray (N191D) below, terminal leaflet 3.0–8.6 by 2.1–4.5 cm, 1.9–2.9 times as long as wide, petioles 0.4–4.7 cm, base attenuate in compound leaves, cuneate to round in unifoliate leaves, terminal petiolules absent, secondary veins 9–12 pairs; lateral leaflets 1.5–6.4 by 0.6–2.5 cm, 1.3–2.7 times as long as wide, secondary veins 6–8 pairs, sessile. Inflorescence terminal, paniculate, 4.9–15.1 cm long axis, seldom with primary branch at panicle base, consisting of lateral dichasial cymes in lax clusters attached to the panicle axis or primary branch, with the oldest central flower often fertilized at maturity; bracteole linear, c. 1 mm. Calyx 5-lobed, accrescent, persistent, with numerous glands; lobes 0.2–0.7 by 0.3–2.4 mm, acute, velutinous; flowering calyx 1.8–2.4 mm long, 1.5–3.2 mm diameter; fruiting calyx 2.5–4.9 mm diameter, covering up to 75% of the mature fruit. Corolla 5-lobed, pale purple, outside covered with appressed hairs; corolla throat inner diameter 2.8–4.0 mm; lower lip orbicular to broadly ovate, 1.9–5.4 by 2.2–5.6 mm, apex acute, margin entire, two clear ridges at corolla mouth, often reflexed, deep violet (N89D) to strong violet (N90A), white at base, with white hairs at corolla mouth; lateral lobes 1.9–2.8 by 2.4–3.1 mm, reflexed, apex rounded, brilliant violet (N92A to N92B); upper lip 2-lobed, lobes 1.8–2.8 by 2.0–2.9 mm, apex acute to rounded; tube infundibular 3.9–6.8 mm long, light violet (N91A to N91B). Stamens slightly didynamous, shorter filaments 3.0–5.8 mm long, longer filaments 4.1–6.1 mm long, inserted c. 1/3 of the way on the corolla tube, exceeding 1.8–3.9 mm from the corolla tube, very light purple (85C); anthers 0.6–1.1 mm long, light violet. Ovary globose, glabrous; style 7.1–9.6 mm long, light violet; stigma 2-lobed, lobes 0.3–0.8 mm long. Fruit when young green with purple tinge, turning brown to black when mature; dried obovoid, 3.4–6.4 by 3.5–6.2 mm, apex truncated, glabrous with numerous glands.

**Figure 18.**
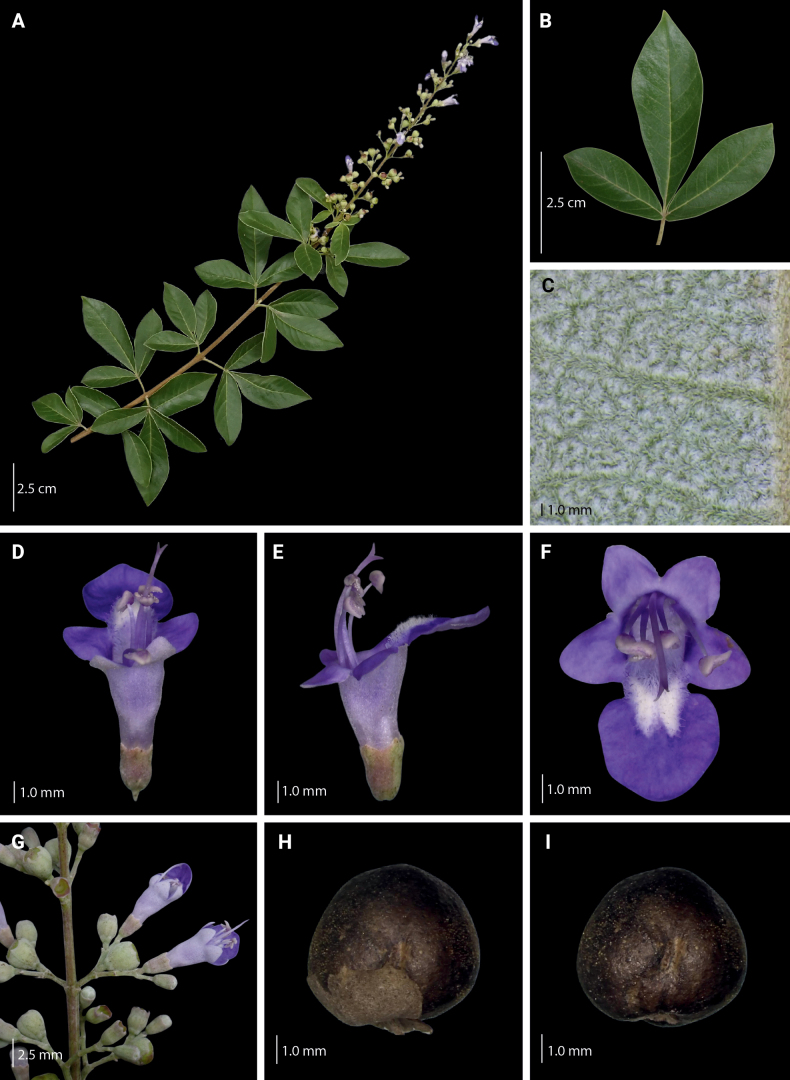
Morphological characteristics of *V.trifolia***A** branch with inflorescence **B** sixth fully unfolded leaf **C** abaxial leaf lamina surface **D–F** flower **G** inflorescence unit **H–I** mature fruit with and without calyx.

###### Phenology.

Flowering and fruiting all year round.

###### Habitat and ecology.

Beaches, but sometimes occurring in thickets and waste places, can also be found in cultivation, particularly in home gardens as both ornamental and medicinal article. Altitude: Usually at sea level but rarely up to 685(–1500) m.

###### Distribution.

Widespread from India and Sri Lanka to Southern Japan and north and east coast of Australia and the Pacific Islands. Indigenous throughout the Philippines.

###### Vernacular names.

lagundi (Tagalog, Bikolano, Bisaya), lagunding-dagat (Tagalog).

###### Notes.

This species is seldom utilized for its medicinal properties.

###### Documented uses (in the Philippines).

Boiling/decoction of leaves for cough and fever; treating chickens; root decoction taken internally for malaria; seeds and fruit for poisonous and venomous animal bites such as snakes.

**Figure 19.**
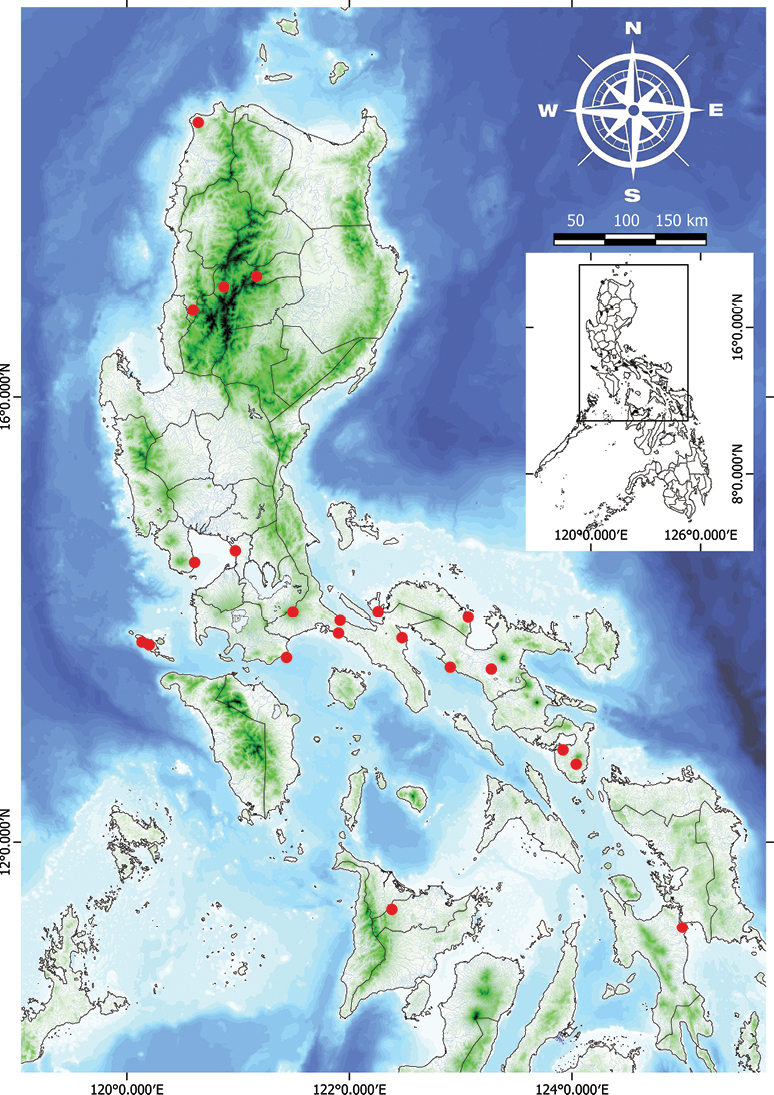
Natural distribution of *Vitextrifolia* in the Philippines.

###### Specimens examined.

**Philippines. Ilocos Norte**: Burgos, Feb–Mar 1917, M. Ramos 27118 (BO1616592); **Ilocos Sur**: Sugpon, Nov 1908, M. L. Merritt & F. W. Darling 14079 (L.2768535); **Mt. Province**: Barlig, Kaleo, Mar 1914, Vanoverbergh 321 (L.2768321); Bauko, 1000–1500 m alt., Vanoberbergh & Skent s.n. (BR0000026253497V); **Bataan**: Limay, Lamao, Oct 1905, H. M. Curran 5295 (BR0000034904909); **Manila**: Tondo, Gagalangin, Loher 4436 (US03805772); **Batangas**: San Juan, Laiya, Oct 1946, M. D. Sulit 7441 (PNH); **Quezon**: Guinayangan, Vidal 850 (L.2768538); Atimonan, Aug 1904, H. N. Whitford 674 (03805776); Lucban, Mt. Banahaw, H. N. Whitford 988 (US03805773); May 1907, Elmer 7877 (US03805766, L.2768533); Calauag, Kinamaligan, E. Canicosa 9784 (PNH); Agdangan, Ibabang Kinagunan, May 1976, R. T. Francisco 170836 (PNH); **Occidental Mindoro**: Lubang, Apr 1903, Merrill 957 (US03805767); Lubang, Tilik, 685 m alt., 5 Jul 1996, Romero & Fuentes 37610 (L.4212394); **Camarines Norte**: Daet, Marcedes, H. Hallier 4230b (L.2768536); **Camarines Sur**: Pasacao, Dalupaon, Ahern 255 (US03805779); Pili, Carambola, May 1947, P. Convocar 2962 (PNH); **Sorsogon**: Irosin, Mt. Bulusan, Dec 1915, Elmer 15236 (L.2768324, U.1762516, US03805771); Mar 1958, M. L. Steiner 1327(PNH); **Capiz**: Jamindan, May 1918, G. Edaño 31523 (L.2768539); **Leyte**: Tacloban, Utap, G. Frohne 39320 (PNH).

## Supplementary Material

XML Treatment for Vitex arvensis

XML Treatment for Vitex bicolor

XML Treatment for Vitex elmeri

XML Treatment for Vitex rotundifolia

XML Treatment for Vitex trifolia

## ﻿Additional information

### Author ORCIDs

Renerio P. Gentallan Jr. https://orcid.org/0000-0002-6436-7878

Seda Sengun https://orcid.org/0000-0003-3126-8707

Michael Cedric B. Bartolome https://orcid.org/0000-0002-3579-1194

Kristine Joyce O. Quiñones https://orcid.org/0000-0002-1709-3317

Nadine B. Coronado https://orcid.org/0000-0002-4951-3830

Teresita H. Borromeo https://orcid.org/0000-0002-8625-4110

Emmanuel Bonifacio S. Timog https://orcid.org/0000-0002-0302-6519

### Data availability

All of the data that support the findings of this study are available in the main text or Supplementary Information.

## Supplementary materials

Supplementary material 1 Additional information Data type xlsx https://binary.pensoft.net/file/1158198This dataset is made available under the Open Database License (http://opendatacommons.org/licenses/odbl/1.0/). The Open Database License (ODbL) is a license agreement intended to allow users to freely share, modify, and use this Dataset while maintaining this same freedom for others, provided that the original source and author(s) are credited.Renerio P. Gentallan Jr., Seda Sengun, Michael Cedric B. Bartolome, Kristine Joyce O. Quiñones, Nadine B. Coronado, Teresita H. Borromeo, Emmanuel Bonifacio S. Timog

Supplementary material 2 Chloroplast genome information Data type docx https://binary.pensoft.net/file/1158199This dataset is made available under the Open Database License (http://opendatacommons.org/licenses/odbl/1.0/). The Open Database License (ODbL) is a license agreement intended to allow users to freely share, modify, and use this Dataset while maintaining this same freedom for others, provided that the original source and author(s) are credited.Renerio P. Gentallan Jr., Seda Sengun, Michael Cedric B. Bartolome, Kristine Joyce O. Quiñones, Nadine B. Coronado, Teresita H. Borromeo, Emmanuel Bonifacio S. Timog

Supplementary material 3 Additional specimens examined Data type docx https://binary.pensoft.net/file/1158200This dataset is made available under the Open Database License (http://opendatacommons.org/licenses/odbl/1.0/). The Open Database License (ODbL) is a license agreement intended to allow users to freely share, modify, and use this Dataset while maintaining this same freedom for others, provided that the original source and author(s) are credited.Renerio P. Gentallan Jr., Seda Sengun, Michael Cedric B. Bartolome, Kristine Joyce O. Quiñones, Nadine B. Coronado, Teresita H. Borromeo, Emmanuel Bonifacio S. Timog
