# Supplementary material for: ﻿The Vitextrifolia complex (Lamiaceae) in the Philippines
Source: PhytoKeys. 2024 Oct 22;248:1–40. doi: 10.3897/phytokeys.248.120387 (PMC11522745; doi:10.3897/phytokeys.248.120387)

1. ***Vitex agnus-castus* (Chase 22221)**


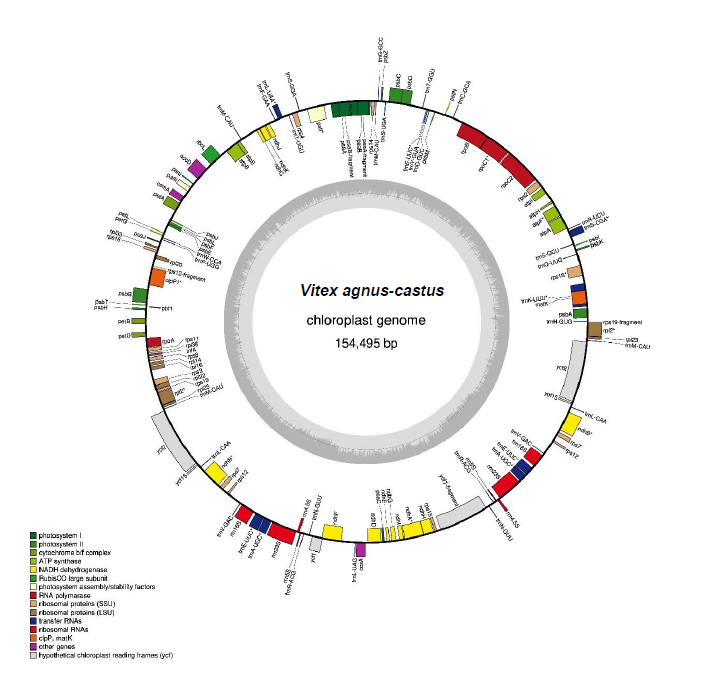

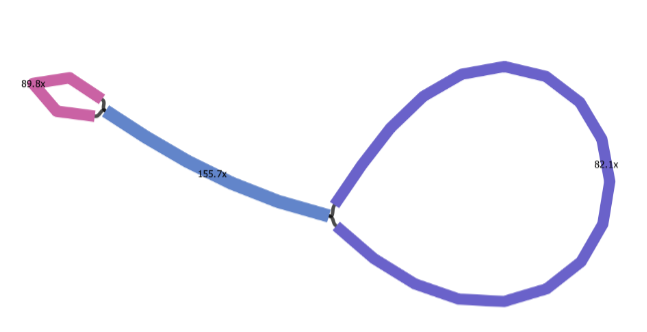


1. ***V. arvensis* (PBN 2019-619)**


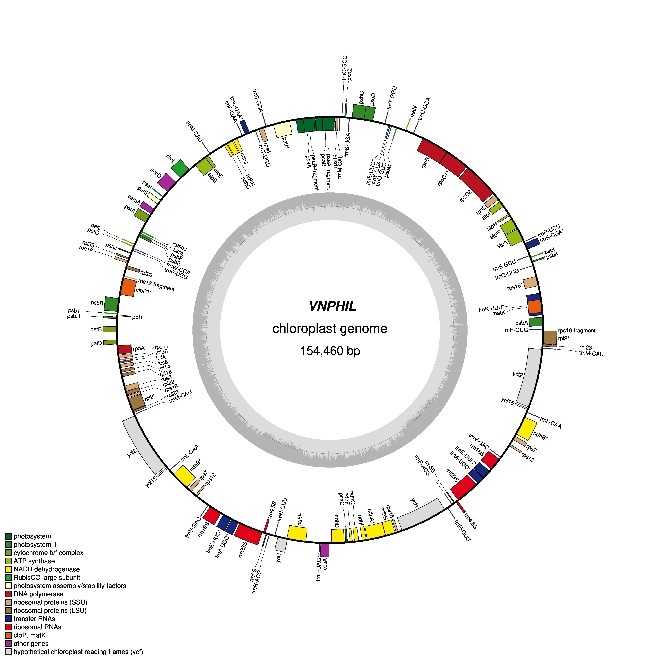


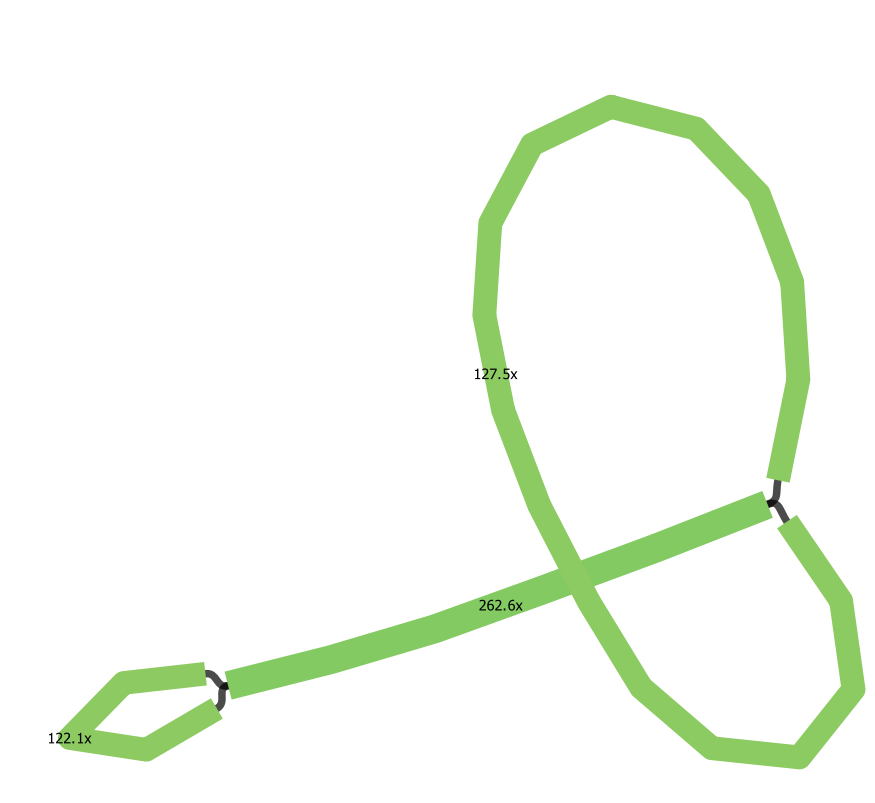


.

1. ***V. arvensis* (PBN 2023-007)**


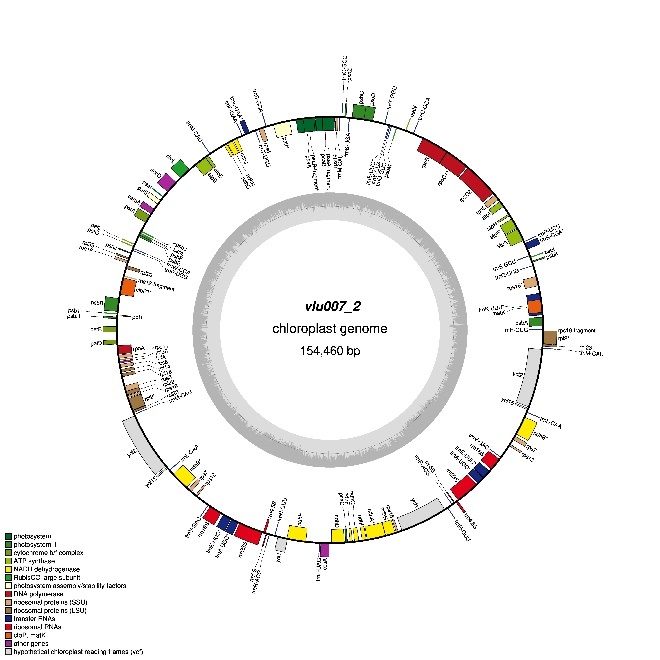


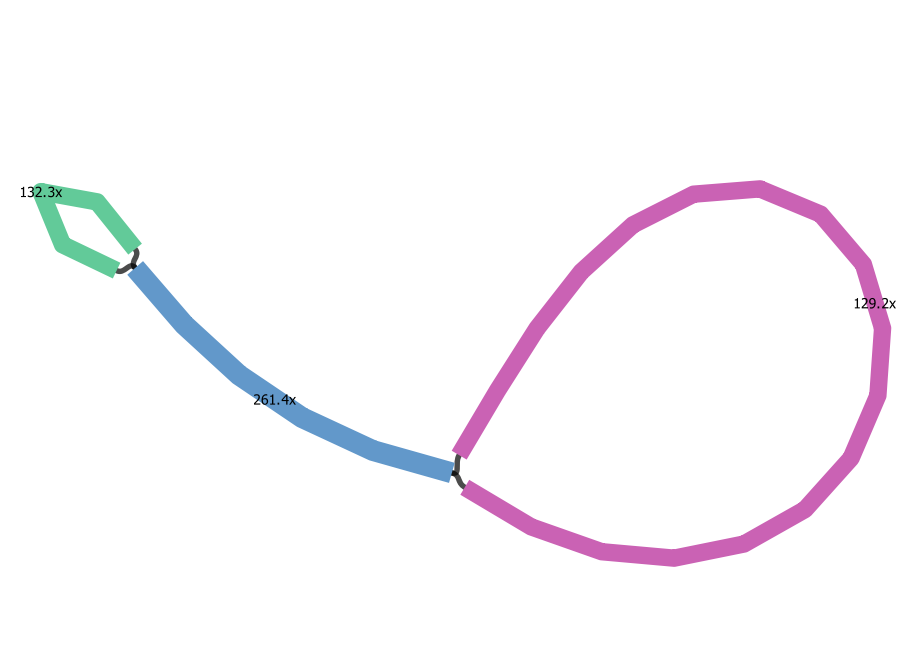


1.
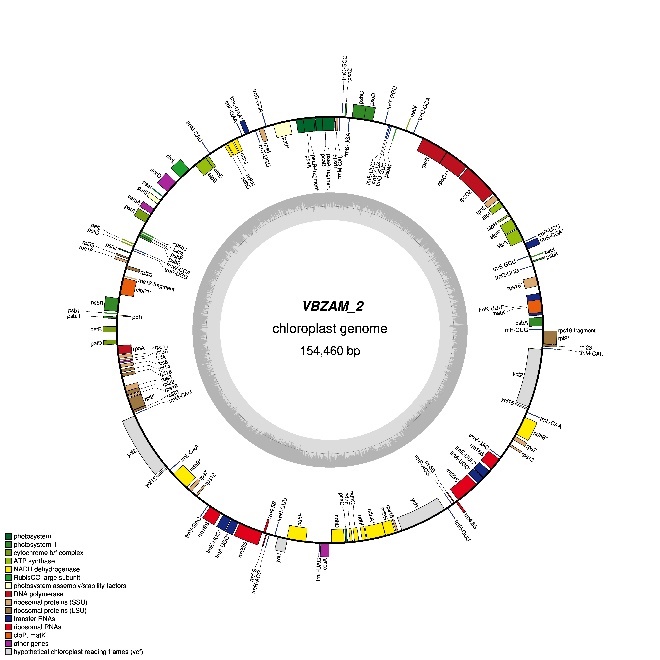
***V. arvensis* (PBN 2019-133)**


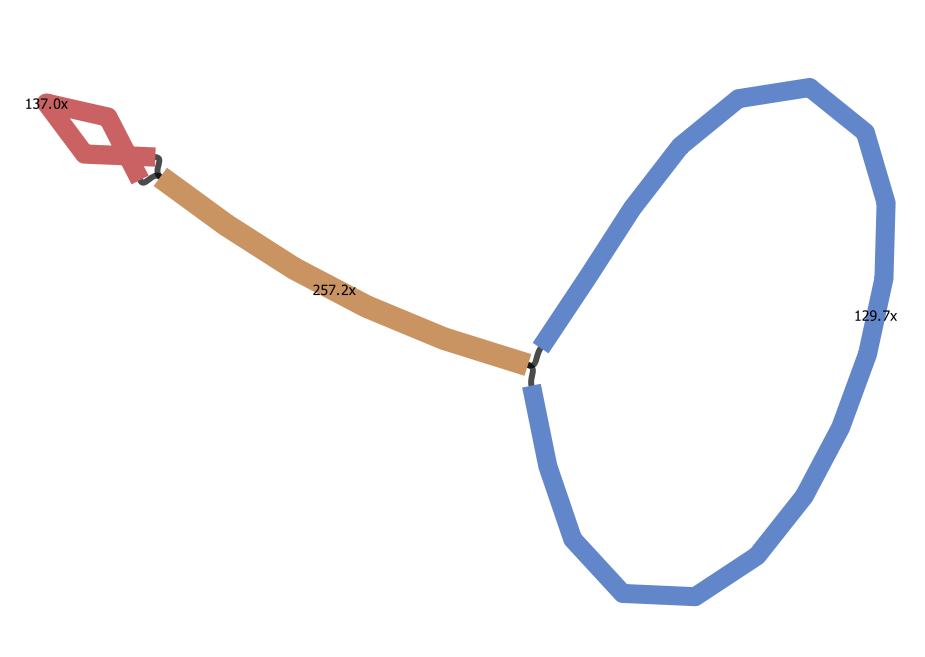


1. ***V. arvensis* (PBN 2019-138)**


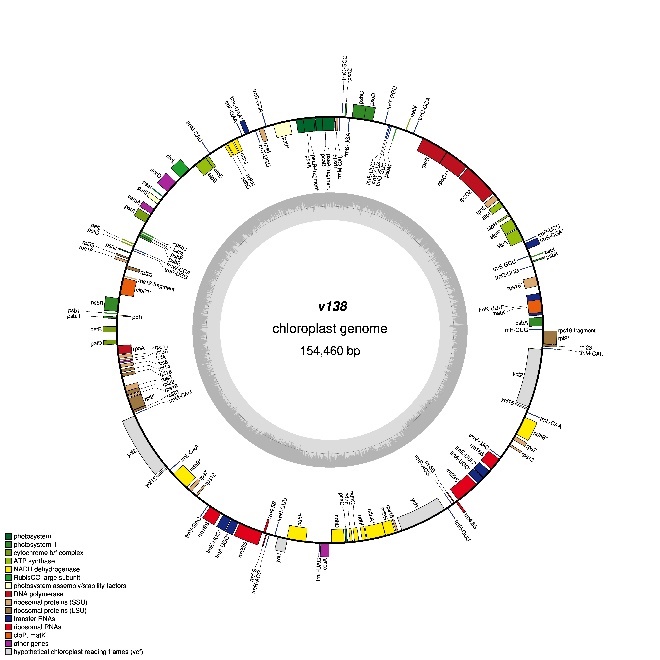


1. ***V. negundo* (Sengun 31)**


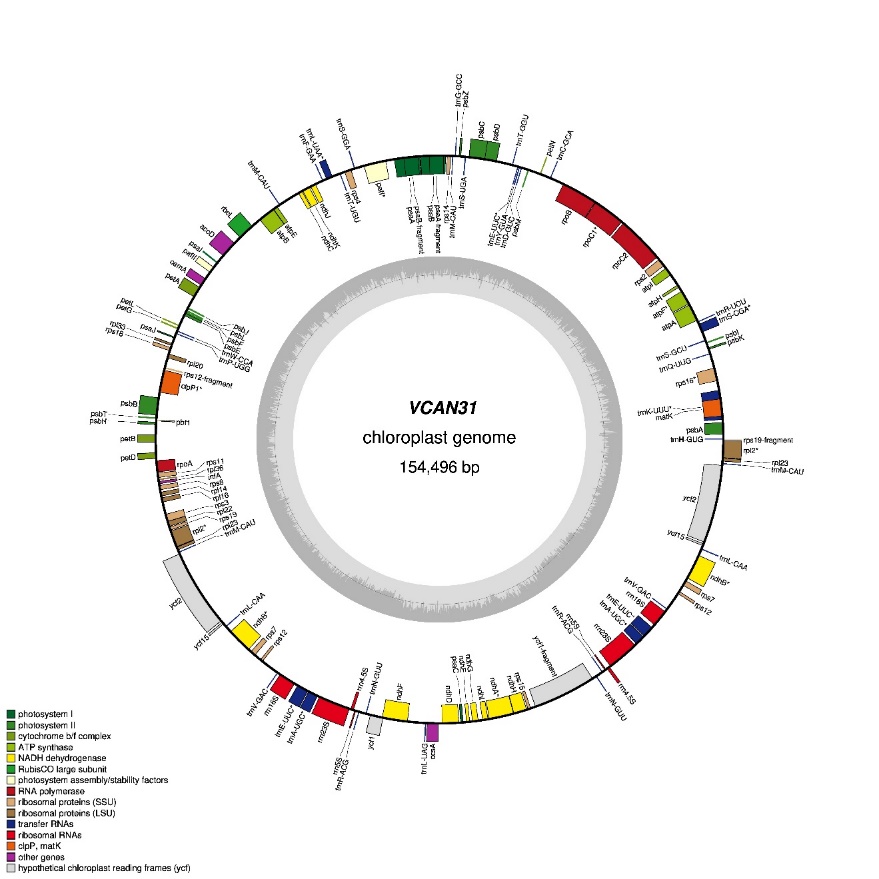


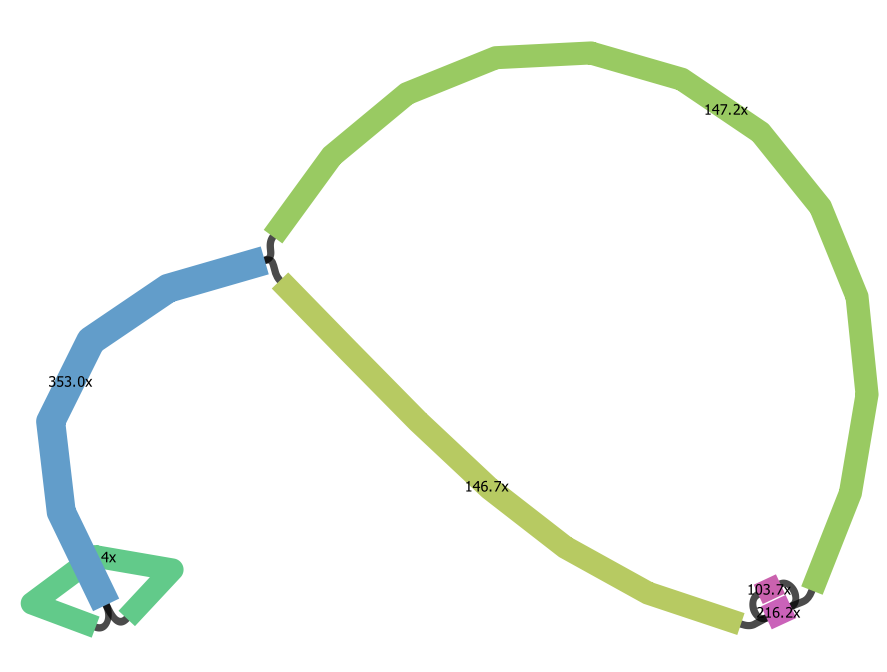


1. ***V. elmeri* (PBN 2018-295)**


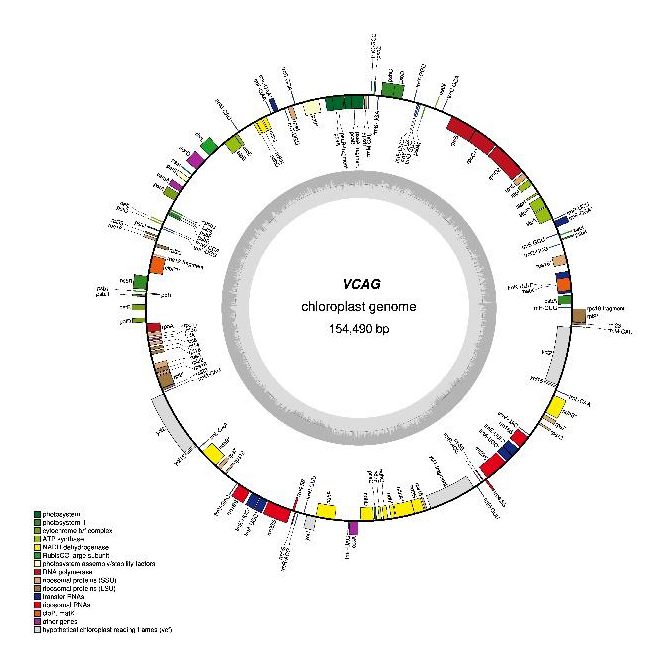


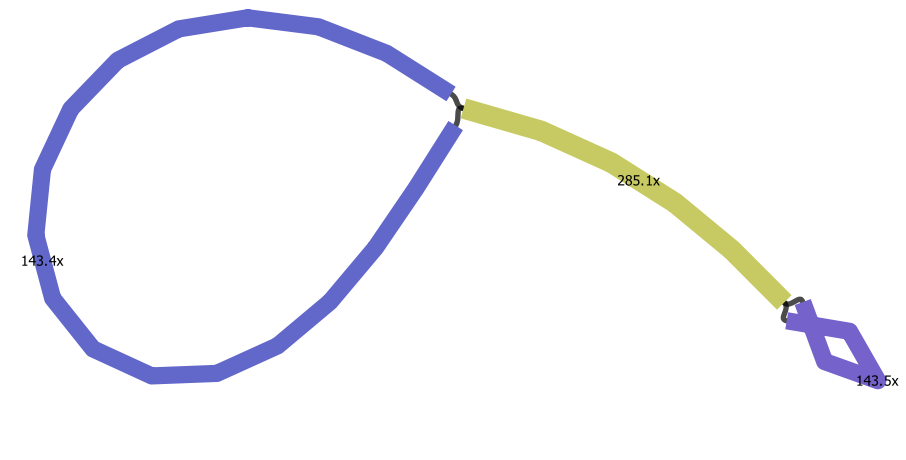


1. ***V. elmeri* (PBN 2023-022)**


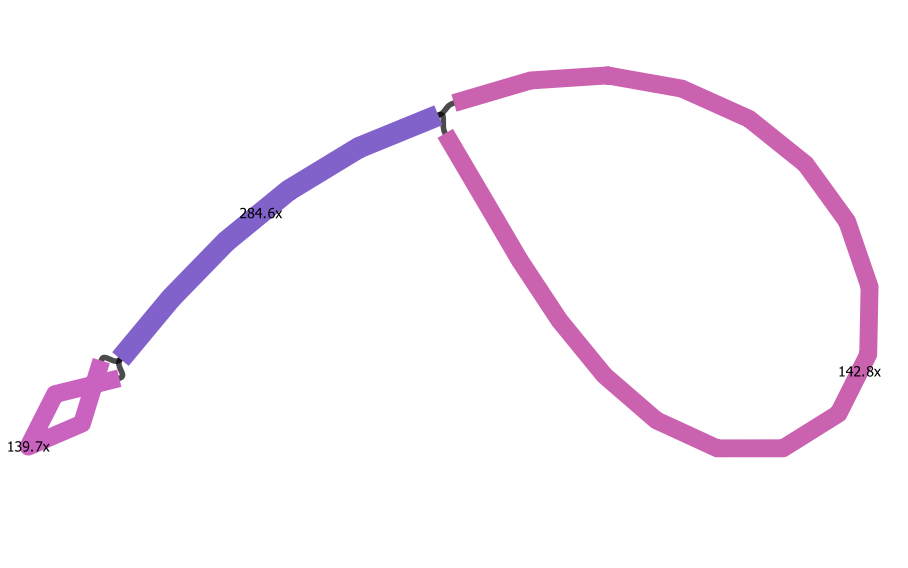


1. ***V. elmeri* (PBN 2018-116)**


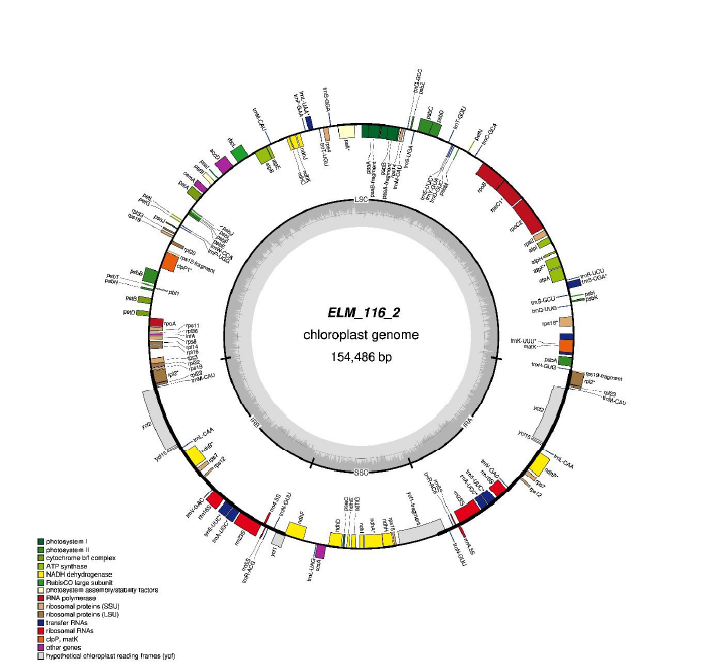


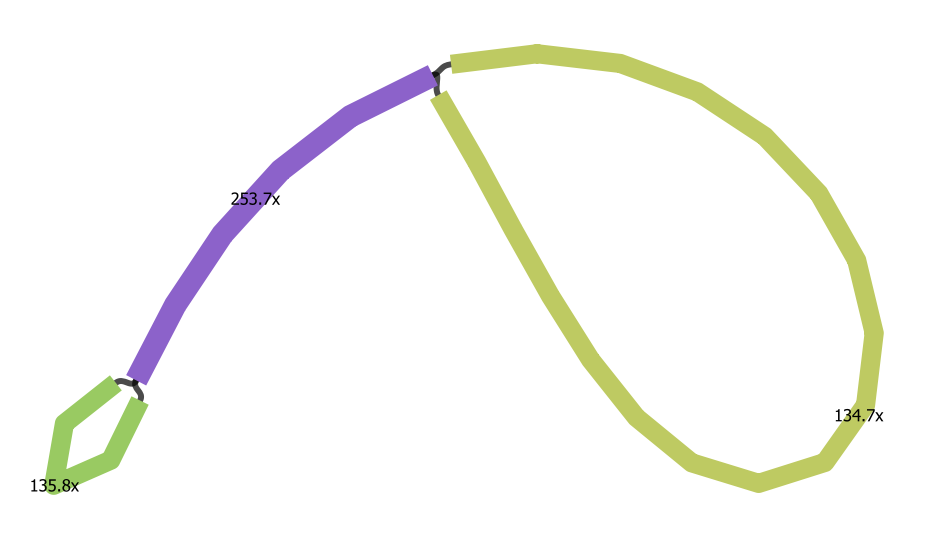


1. ***V. elmeri* (PBN 2018-132)**


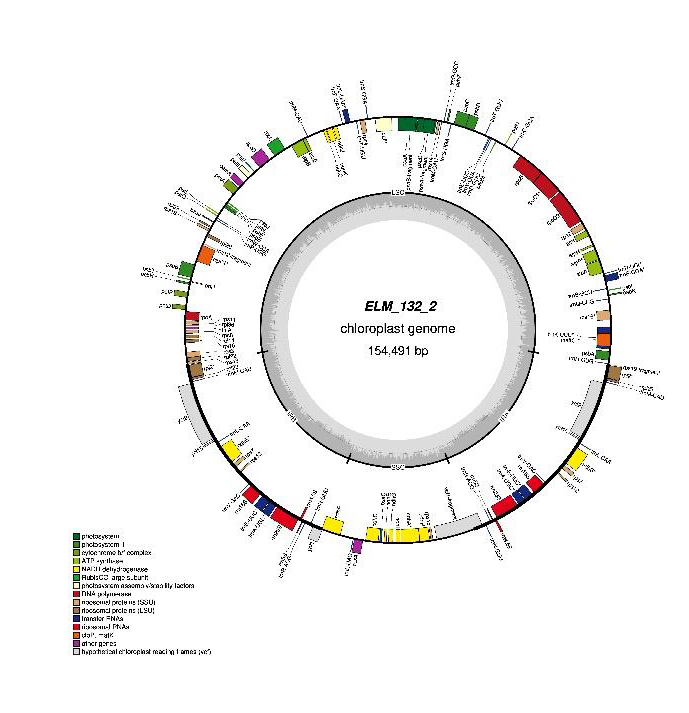


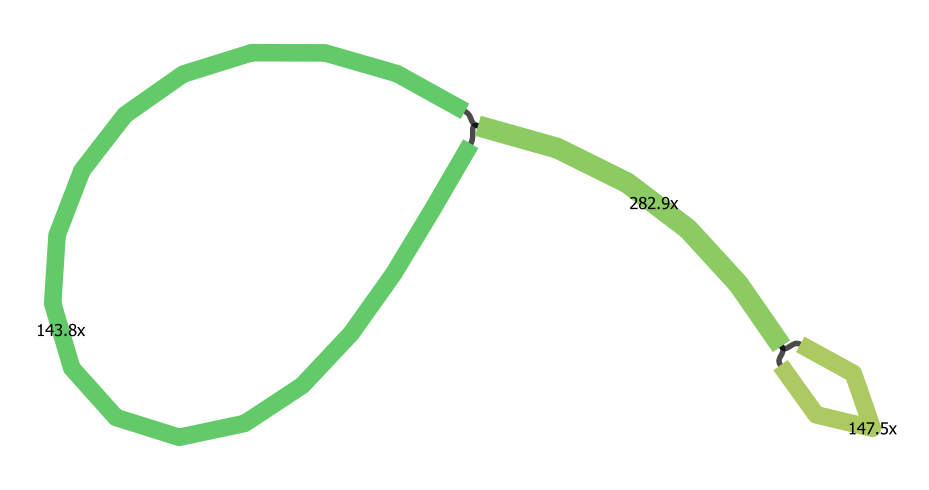


1. ***V. elmeri* (PBN 2019-177)**


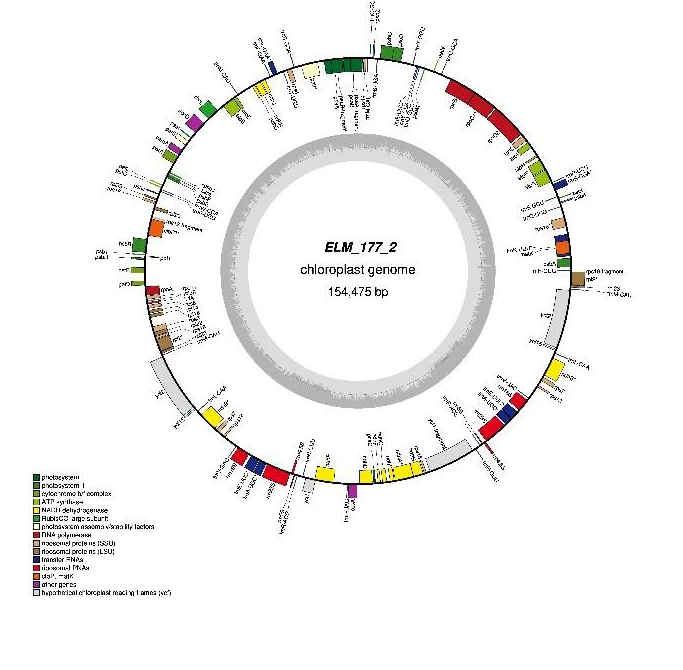


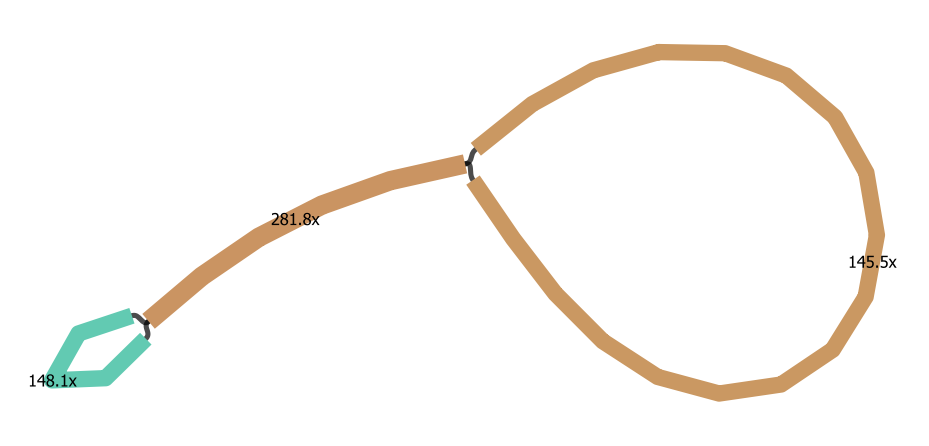


1. ***V. elmeri* (PBN 2018-146)**


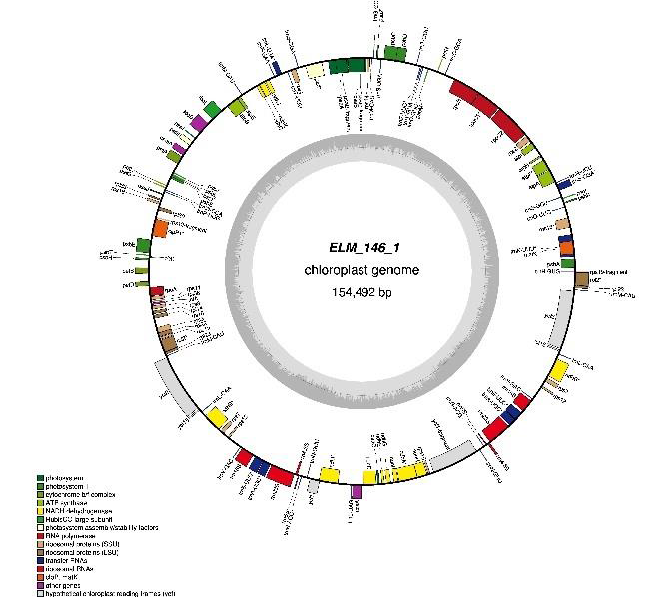


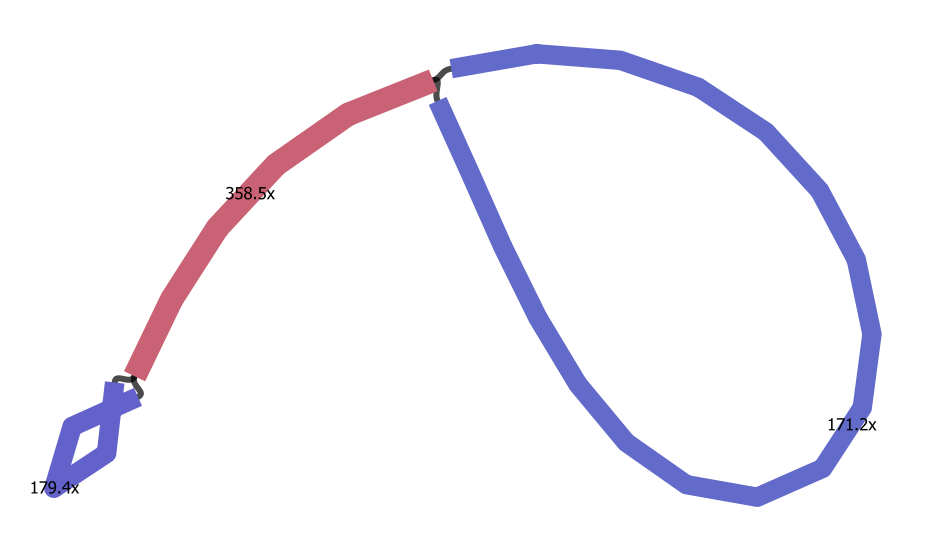


1. ***V. bicolor* (PBN 2018-674)**


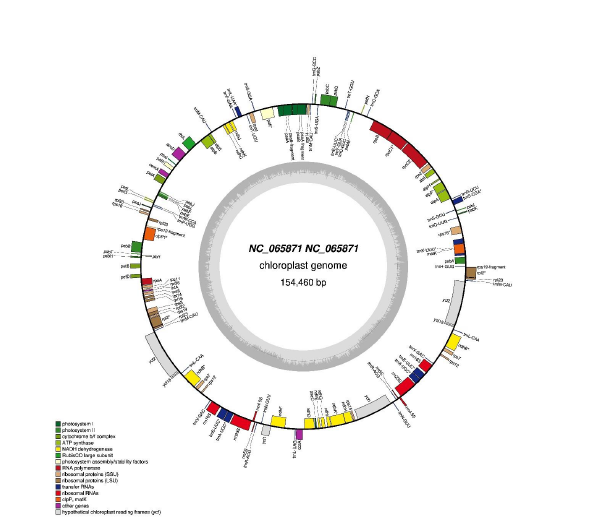


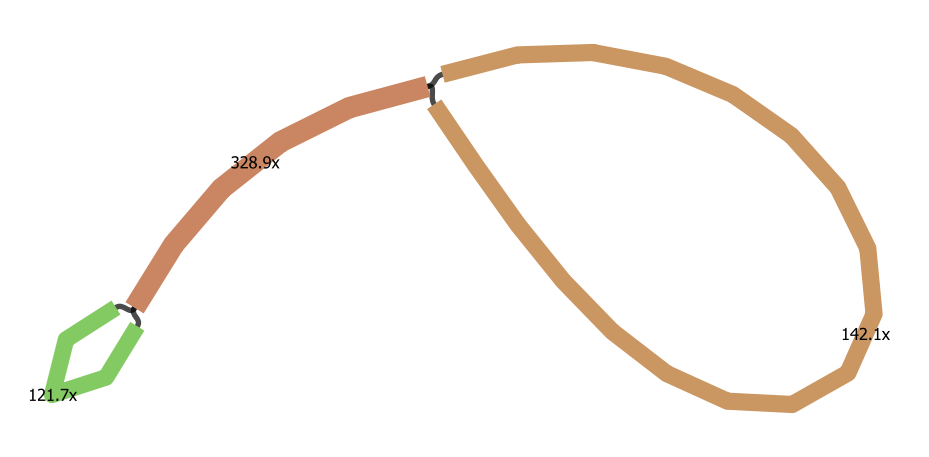


1. ***V. rotundifolia* (PBN 2021-033)**


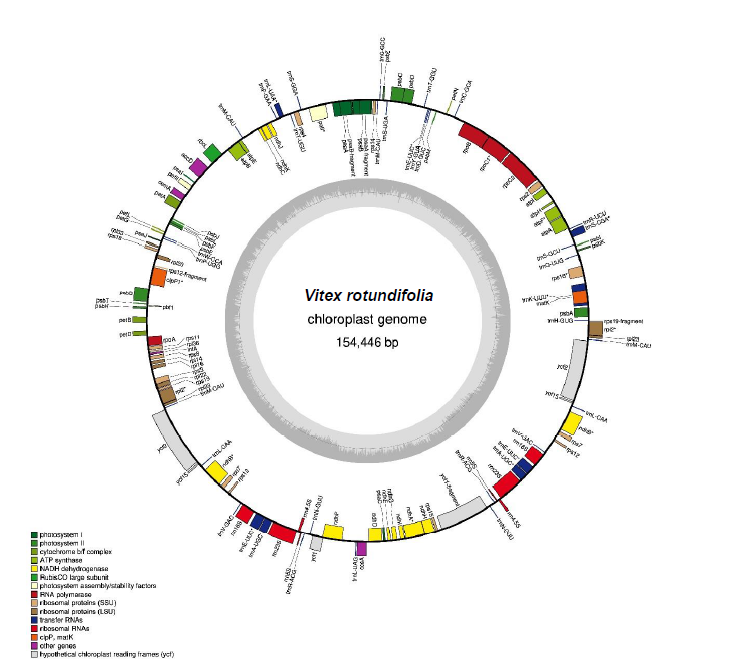


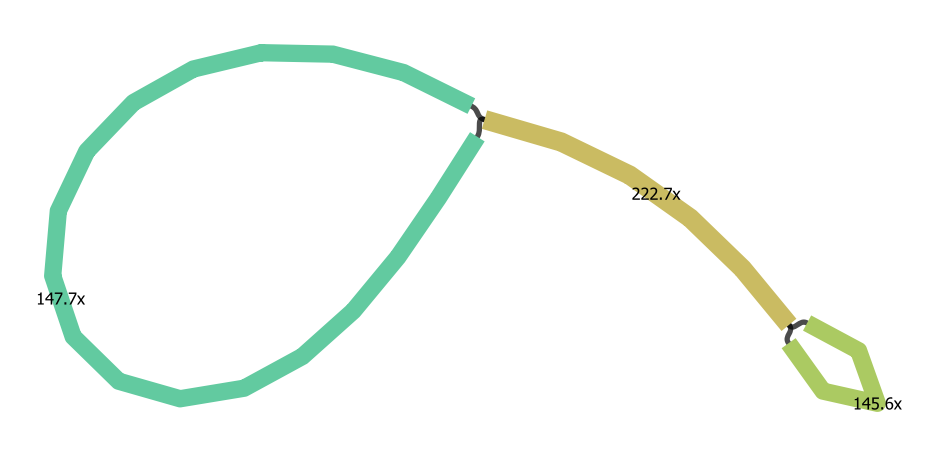


1. ***V. trifolia* (PBN 2019-332)**


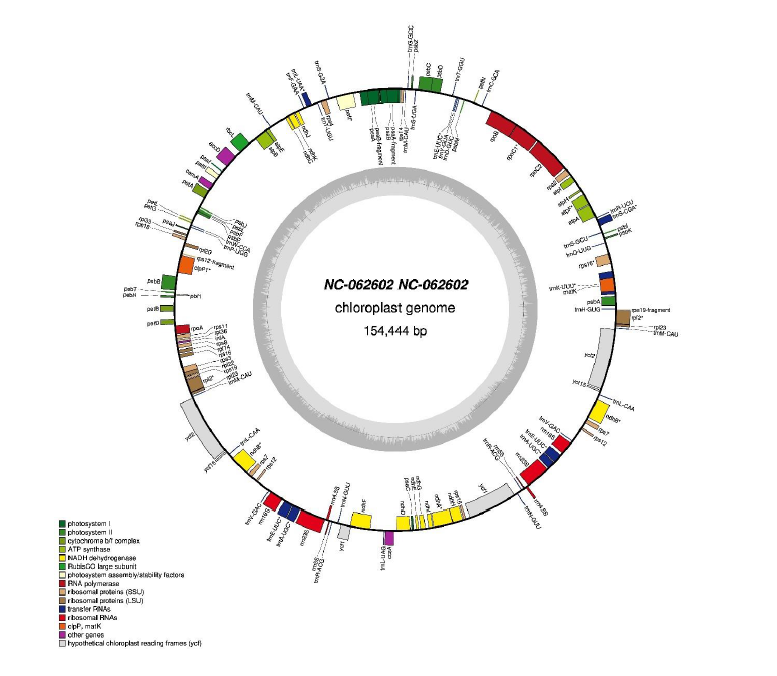


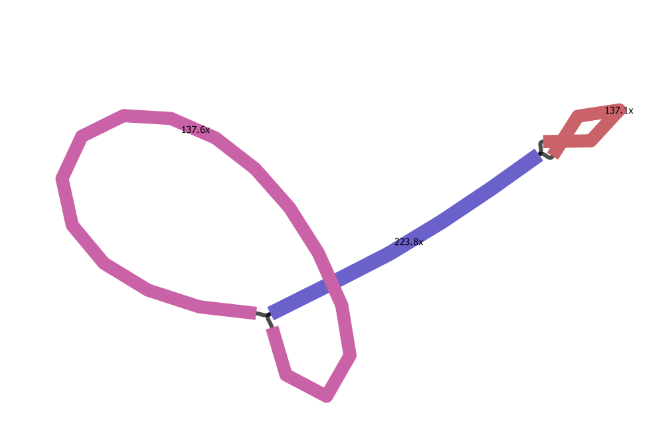


1. ***V. parviflora* (ICROPS 1373)**


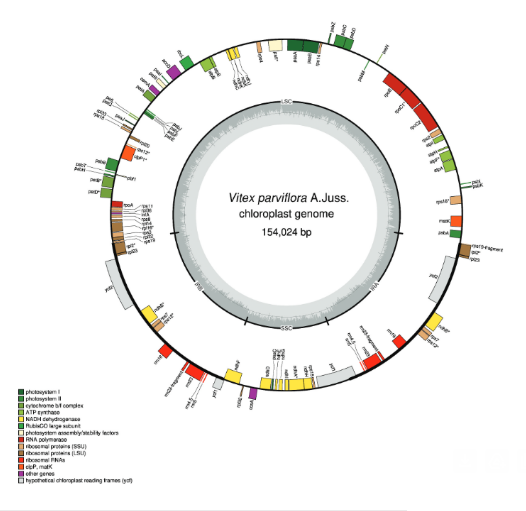


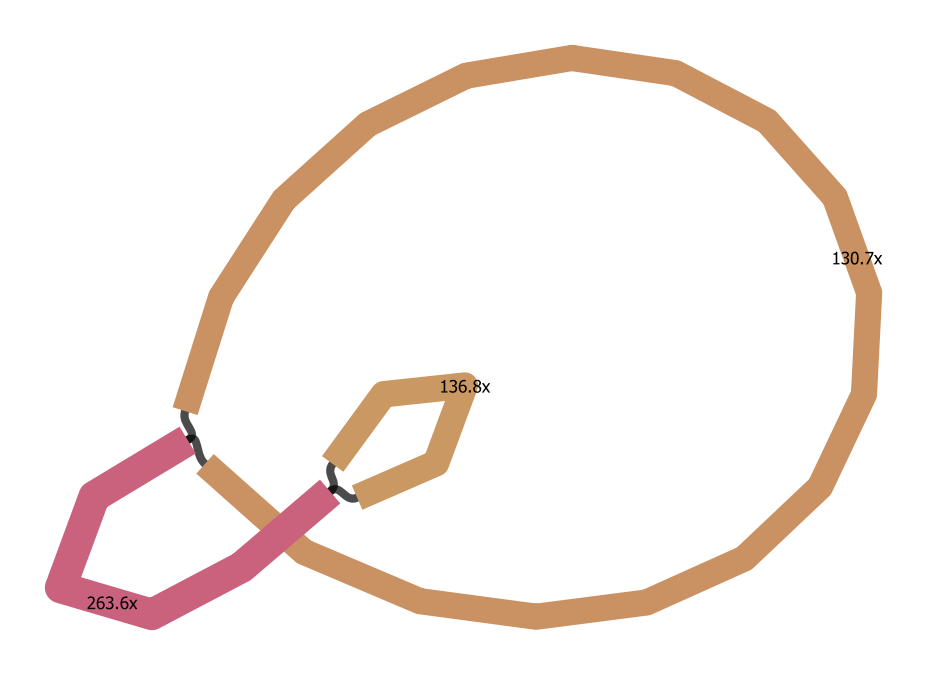

Supplement: Supplementary material 2 — Chloroplast genome information [file phytokeys-248-001_article-120387__-s002.docx]
