# Supplementary material for: ﻿The Vitextrifolia complex (Lamiaceae) in the Philippines
Source: PhytoKeys. 2024 Oct 22;248:1–40. doi: 10.3897/phytokeys.248.120387 (PMC11522745; doi:10.3897/phytokeys.248.120387)
Supplement: Supplementary material 3 — Additional specimens examined [file phytokeys-248-001_article-120387__-s003.docx]

**Supplementary Material 3.** Additional specimens examined

| Herbarium Code | Collector No. | Species | Municipality | Province |
| --- | --- | --- | --- | --- |
| ICROPS1481 | Gentallan & Bartolome 899 | *V. arvensis* | Bolaney | Alaminos |
| ICROPS1511 | Gentallan & Bartolome 1387 | *V. arvensis* | Kabugao | Apayao |
| ICROPS1512 | Gentallan & Bartolome 1406 | *V. arvensis* | Flora | Apayao |
| ICROPS1513 | Gentallan & Bartolome 1416 | *V. arvensis* | Luna | Apayao |
| ICROPS1514 | Gentallan & Bartolome 1427 | *V. arvensis* | Zumigui | Apayao |
| ICROPS1485 | Gentallan & Bartolome 972 | *V. arvensis* | Sta. Ana | Cagayan |
| ICROPS1486 | Gentallan & Bartolome 983 | *V. arvensis* | Sta. Ana | Cagayan |
| ICROPS1487 | Gentallan & Bartolome 998 | *V. arvensis* | Aparri | Cagayan |
| ICROPS1488 | Gentallan & Bartolome 1006 | *V. arvensis* | Toran | Cagayan |
| ICROPS1477 | Gentallan & Bartolome 815 | *V. arvensis* | Dinalupihan | Bataan |
| ICROPS1478 | Gentallan & Bartolome 816 | *V. arvensis* | Dinalupihan | Bataan |
| ICROPS1469 | Gentallan & Bartolome 360 | *V. arvensis* | Sta. Maria | Laguna |
| ICROPS1476 | Gentallan & Bartolome 743 | *V. arvensis* | Los Baños | Laguna |
| ICROPS1503 | Gentallan & Bartolome 1261 | *V. arvensis* | Bulalacao | Oriental Mindoro |
| ICROPS1504 | Gentallan & Bartolome 1274 | *V. arvensis* | Calapan City | Oriental Mindoro |
| ICROPS1502 | Gentallan & Bartolome 1239 | *V. arvensis* | Sablayan | Occidental Mindoro |
| ICROPS1470 | Gentallan & Bartolome 424 | *V. arvensis* | Sto. Domingo | Albay |
| ICROPS1495 | Gentallan & Bartolome 1161 | *V. arvensis* | Altavas | Aklan |
| ICROPS1494 | Gentallan & Bartolome 1142 | *V. arvensis* | Sebaste | Antique |
| ICROPS1492 | Gentallan & Bartolome 1086 | *V. arvensis* | Sigma | Capiz |
| ICROPS1490 | Gentallan & Bartolome 1050 | *V. arvensis* | Passi | Iloilo |
| ICROPS1491 | Gentallan & Bartolome 1069 | *V. arvensis* | San Enrique | Iloilo |
| ICROPS1497 | Gentallan & Bartolome 1186 | *V. arvensis* | Leon | Iloilo |
| ICROPS1498 | Gentallan & Bartolome 1200 | *V. arvensis* | Tigbauan | Iloilo |
| ICROPS1505 | Gentallan & Bartolome 1293 | *V. arvensis* | Tagbilaran City | Bohol |
| ICROPS1506 | Gentallan & Bartolome 1311 | *V. arvensis* | Guindulman | Bohol |
| ICROPS1508 | Gentallan & Bartolome 1335 | *V. arvensis* | Antenquera | Bohol |
| ICROPS1509 | Gentallan & Bartolome 1348 | *V. arvensis* | Calape | Bohol |
| ICROPS474 | Gentallan & Bartolome 714 | *V. arvensis* | Dolores | Eastern Samar |
| ICROPS1475 | Gentallan & Bartolome 721 | *V. arvensis* | Taft | Eastern Samar |
| ICROPS1471 | Gentallan & Bartolome 481 | *V. arvensis* | Baybay City | Leyte |
| ICROPS1463 | Gentallan & Bartolome 198 | *V. arvensis* | Cagayan de Oro City | Misamis Oriental |
| ICROPS1464 | Gentallan & Bartolome 254 | *V. arvensis* | Cagayan de Oro City | Misamis Oriental |
| ICROPS1465 | Gentallan & Bartolome 275 | *V. arvensis* | Valencia City | Bukidnon |
| ICROPS1466 | Gentallan & Bartolome 283 | *V. arvensis* | Dabong-Dabong | Bukidnon |
| ICROPS1467 | Gentallan & Bartolome 316 | *V. arvensis* | Malitbog | Bukidnon |
| ICROPS1468 | Gentallan & Bartolome 322 | *V. arvensis* | Malitbog | Bukidnon |
| ICROPS1456 | Gentallan & Bartolome 086 | *V. bicolor* | Lobo | Batangas |
| ICROPS1472 | Gentallan & Bartolome 574 | *V. bicolor* | Siquijor | Siquijor |
| ICROPS1458 | Gentallan & Bartolome 123 | *V. elmeri* | San Nicolas | Ilocos Norte |
| ICROPS1461 | Gentallan & Bartolome 161 | *V. elmeri* | Kamandingan | Ilocos Norte |
| ICROPS 1462 | Gentallan & Bartolome 162 | *V. elmeri* | Kamandingan | Ilocos Norte |
| ICROPS1510 | Gentallan & Bartolome 1385 | *V. elmeri* | Conner | Apayao |
| ICROPS1483 | Gentallan & Bartolome 927 | *V. elmeri* | Sanchez Mira | Cagayan |
| ICROPS1484 | Gentallan & Bartolome 949 | *V. elmeri* | Buguey | Cagayan |
| ICROPS1486 | Gentallan & Bartolome 983 | *V. elmeri* | Sta. Ana | Cagayan |
| ICROPS1499 | Gentallan & Bartolome 1217 | *V. elmeri* | Mamburao | Occidental Mindoro |
| ICROPS1500 | Gentallan & Bartolome 1221 | *V. elmeri* | Mamburao | Occidental Mindoro |
| ICROPS1501 | Gentallan & Bartolome 1233 | *V. elmeri* | Sablayan | Occidental Mindoro |
| ICROPS1493 | Gentallan & Bartolome 1120 | *V. elmeri* | Tibiao | Antique |
